# Cross-Sectional and Longitudinal MRI Brain Scans Reveal Accelerated Brain Aging in Multiple Sclerosis

**DOI:** 10.3389/fneur.2019.00450

**Published:** 2019-04-30

**Authors:** Einar A. Høgestøl, Tobias Kaufmann, Gro O. Nygaard, Mona K. Beyer, Piotr Sowa, Jan E. Nordvik, Knut Kolskår, Geneviève Richard, Ole A. Andreassen, Hanne F. Harbo, Lars T. Westlye

**Affiliations:** ^1^Institute of Clinical Medicine, University of Oslo, Oslo, Norway; ^2^NORMENT, Division of Mental Health and Addiction, Oslo University Hospital & Institute of Clinical Medicine, University of Oslo, Oslo, Norway; ^3^Department of Neurology, Oslo University Hospital, Oslo, Norway; ^4^Division of Radiology and Nuclear Medicine, Oslo University Hospital, Oslo, Norway; ^5^Catosenteret Rehabilitation Centre, Son, Norway; ^6^Sunnaas Rehabilitation Hospital HT, Nesodden, Norway; ^7^Department of Psychology, University of Oslo, Oslo, Norway

**Keywords:** multiple sclerosis, brain age, magnetic resonance imaging, machine learning, longitudinal

## Abstract

Multiple sclerosis (MS) is an inflammatory disorder of the central nervous system. By combining longitudinal MRI-based brain morphometry and brain age estimation using machine learning, we tested the hypothesis that MS patients have higher brain age relative to chronological age than healthy controls (HC) and that longitudinal rate of brain aging in MS patients is associated with clinical course and severity. Seventy-six MS patients [71% females, mean age 34.8 years (range 21–49) at inclusion] were examined with brain MRI at three time points with a mean total follow up period of 4.4 years (±0.4 years). We used additional cross-sectional MRI data from 235 HC for case-control comparison. We applied a machine learning model trained on an independent set of 3,208 HC to estimate individual brain age and to calculate the difference between estimated and chronological age, termed brain age gap (BAG). We also assessed the longitudinal change rate in BAG in individuals with MS. MS patients showed significantly higher BAG (4.4 ± 6.6 years) compared to HC (Cohen's D = 0.69, *p* = 4.0 × 10^−6^). Longitudinal estimates of BAG in MS patients showed high reliability and suggested an accelerated rate of brain aging corresponding to an annual increase of 0.41 (SE = 0.15) years compared to chronological aging (*p* = 0.008). Multiple regression analyses revealed higher rate of brain aging in patients with more brain atrophy (Cohen's D = 0.86, *p* = 4.3 × 10^−15^) and increased white matter lesion load (WMLL) (Cohen's D = 0.55, *p* = 0.015). On average, patients with MS had significantly higher BAG compared to HC. Progressive brain aging in patients with MS was related to brain atrophy and increased WMLL. No significant clinical associations were found in our sample, future studies are warranted on this matter. Brain age estimation is a promising method for evaluation of subtle brain changes in MS, which is important for predicting clinical outcome and guide choice of intervention.

## Introduction

Multiple sclerosis (MS) is an inflammatory, demyelinating disease of the CNS. The pathophysiology of MS can be divided into acute inflammation during a relapse and chronic inflammation thought to continuously perturb neuroaxonal homeostasis and drive neurodegeneration (1). Development of robust brain imaging markers that can parse between-subject heterogeneity of the clinical trajectories, predict future progression of disability, and monitor the effects of treatment for MS patients, is a major aim with important clinical implications (2, 3). Current imaging markers with relevance for MS are associated with disease activity and progression, and include, among other features, number or volume of hyperintense brain lesions visible on T2-weighted MRI images, contrast-enhancing T1 lesions, increased annual brain volume loss and T1-hypointense “black holes” (2, 4, 5). Increased rate of total brain volume loss, which is best captured using longitudinal designs (6), reflects accelerated neurodegeneration (7), and regional analyses may boost the correlations between estimated brain atrophy and disability (2).

However, identifying robust associations between clinical outcomes and MRI measures has been challenging (8). This clinico-radiological paradox in MS is likely explained by a combination of lack of sensitivity and specificity both in the clinical and imaging domain. Brain age estimation uses machine learning to train a model that can accurately predict the individual age from brain imaging data (9–11). Utilizing sensitive measures of MRI-based brain morphometry, brain age estimation provides a robust imaging-based biomarker with potential to yield novel insights into similarities and differences of disease pathophysiology across brain disorders (11, 12). Such imaging-based brain age has been shown to be reliable both within and between MRI scanners, and is a candidate biomarker of an individual's brain health and integrity (10–12). Different approaches to brain age estimation exploit information from a variety of brain regions (e.g., hippocampus, subcortical, gray matter, and white matter) or MRI sequences (e.g., T1, T2, diffusion tensor imaging and functional MRI) to inform the model (12). An older appearing brain, which is related to advanced physiological and cognitive aging and mortality (12, 13), has been found across several brain disorders, and region specific brain age patterns in patient cohorts have shown potential differential genetic effects, including genetic pleiotropy between global brain age and MS (11). To our knowledge, only two preprint manuscripts (11, 14) and one abstract (15) have reported brain age estimations in MS, and all reported older appearing brains in patients with MS compared to HC.

Here, combining cross-sectional and sensitive measures of MRI-based regional and global brain morphometry in MS and HC (cross-sectional only), we tested the hypothesis that MS patients have higher brain age than HC. Next, using longitudinal MRI data in MS patients we tested the hypothesis that brain aging accelerates in MS and that the rate of acceleration is associated with a more severe clinical outcome.

## Materials and Methods

### Participants

We recruited 76 MS patients at Oslo University Hospital (16, 17). All patients were diagnosed with MS between January 2009 and December 2012 according to the revised McDonald Criteria (18) and were enrolled in the study on average 14 months (±11.8) after the date of diagnosis (time point 1). Exclusion criteria included age < 18 years or > 50 years, uncertain diagnosis, non-fluency in Norwegian, neurological or psychiatric disease, drug abuse, head trauma, pregnancy, and previous adverse gadolinium reaction. Most patients also participated in two follow-up examinations on average 26 months (±11.7, time point 2, *n* = 60) and 66 months (±13.3, time point 3, *n* = 62) after the date of diagnosis. At each visit, all patients completed a neurological examination by a Neurostatus certified medical doctor (http://www.neurostatus.com) within the same week as their MRI scan. Disease-modifying treatments were categorized into the following groups; 0: no treatment; 1: glatiramer acetate, interferons, teriflunomide, or dimetylfumarate; and 2: fingolimod, natalizumab, or alemtuzumab. Many patients (*n* = 58) were also included in a partly overlapping study with a larger cross-sectional MS group (*n* = 254) (11).

The HC group was recruited through newspaper ads or after a stratified random selection drawn from the Norwegian National Population Registry to two parallel studies (13, 19). Exclusion criteria included estimated IQ (intelligence quotient) <70, history of neurologic or psychiatric disease and current medication significantly affecting the nervous system (20).

This study was carried out in accordance with the recommendations of the Regional Committee for Medical and Health Research Ethics with written informed consent from all subjects. All subjects gave written informed consent in accordance with the Declaration of Helsinki. The protocol was approved by the South East Regional Committee for Medical and Health Research Ethics.

### MRI Acquisition

All MS patients were scanned at up to three time points between January 2012 and August 2017 in a study setting, using the same 1.5 T scanner (Avanto, Siemens Medical Solutions; Erlangen, Germany) equipped with a 12-channel head coil. Structural MRI data were collected using a 3D T1-weighted MPRAGE (Magnetization Prepared Rapid Gradient Echo) sequence, with the following parameters: TR (repetition time)/TE (echo time)/flip angle/voxel size/FOV (field of view)/slices/scan time/matrix/time to inversion = 2,400 ms/3.61 ms/8°/1.20 × 1.25 × 1.25 mm/240/160 sagittal slices/7:42 min/192 × 192/1,000 ms. The MRI sequence was kept identical during the scanning period. FLAIR (Fluid attenuation inversion recovery), T2 and pre- and post-gadolinium 3D T1 sequences were attained and used for neuroradiological evaluation (17).

Fifty-eight of the MS patients were also scanned at Oslo University Hospital on a 3 T GE 750 Discovery MRI scanner with a 32-channel head coil at time point 3 between August 2016 and June 2017 during the same week they were scanned at the 1.5 T scanner for time point 3. HCs were scanned solely on the 3 T scanner at one time point to provide cross-sectional data. Structural MRI data were collected using a 3D high-resolution IR-prepared FSPGR (fast spoiled gradient echo) T1-weighted sequence (3D BRAVO) with the following parameters: TR (repetition time)/TE (echo time)/flip angle/voxel size/FOV (field of view)/slices/scan time = 8.16 ms/3.18 ms/12°/1 × 1 × 1 mm/256 × 256 mm/188 sagittal slices/4:42 min.

### MRI Pre- and Post-processing

Using the T1-weighted scans we performed cortical reconstruction and volumetric segmentation with FreeSurfer 5.3 (http://surfer.nmr.mgh.harvard.edu/) (21). To extract reliable volume and thickness estimates, images included in the longitudinal 1.5 T MRI dataset were processed with the longitudinal stream in FreeSurfer (22). Specifically an unbiased within-subject template space and image was created using robust, inverse consistent registration (23). Several processing steps, such as skull stripping, Talairach transforms, atlas registration as well as spherical surface maps and parcellations were then initialized with common information from the within-subject template, increasing reliability and power (22).

Manual quality control of the MRI scans from patients was performed by trained research personnel to identify and edit segmentation errors where possible (*n* = 43 MRI scans) and exclude data of insufficient quality (*n* = 6 MRI scans). In addition, eight brain scans were removed due to missing sequences of the 263 MRI scans from MS patients. Lesion filling was performed utilizing automatically generated lesion masks from Cascade (24) with the lesion filling tool (https://fsl.fmrib.ox.ac.uk/fsl/fslwiki/lesion_filling) in FSL (25). The lesion masks were assessed by a trained neuroradiologist and normalized to MNI space using FLIRT (26), with the corresponding T1 image as an intermediate. A probabilistic representation of the lesions across all patients is shown in Supplementary Figure 1.

### Brain Age Estimation Model

The training set for brain age estimation included MRI scans from 3,208 HC >12 years (54% women, mean age 47.5 (±19.8), age range 12–95) obtained from several publicly available datasets (Supplementary Figure 2) and processed in the same MRI pipeline.

We trained one machine learning model for each sex to predict brain age following a recent implementation (11). The features were derived from the Human Connectome Project parcellation of the cortex (27), comprising 180 regions of interest per hemisphere for thickness, area, and volume, respectively. In addition, we used subcortical and cerebellar parcellations from Freesurfer. The full set comprised 1,118 features in total. We used extreme gradient boosting, “xgboost” package in R (28), as the main method for our brain age studies as it has been the lead solution on many machine learning competitions in the field and due to our data being highly monotonic. We compared xgboost to shrinkage linear models (https://cran.r-project.org/package=care) and found converging results, although xgboost performed slightly better in our data (Supplementary Table 1). We trained one extreme gradient boosting tree machine learning model per sex on the training set to predict age using the 28 brain imaging features (learning rate eta = 0.01, optimal number of rounds determined in a nested cross-validation loop within the training set, other parameters as default). A 10-fold cross-validation confirmed good performance and generalizability in the combined model for females and males (Supplementary Figure 3, *r* = 0.91).

Next, for all patients and HC in the test set, we estimated brain age and calculated the brain age gap (BAG, defined as the difference between chronological age and imaging-based brain age). Using linear regressions, we removed any common variance with age, age^2^ and sex to account for confounding factors before submitting the residualized version of BAG to further analyses (29). When pooling estimates of BAG from the 1.5 T and 3 T scanners, we adjusted BAG for scanner effect on BAG estimates by extracting the scanner coefficient from a LME (linear mixed effects) model. When comparing BAG between patients and matched HCs we report the actual adjusted difference in BAG between these two groups.

In addition to the estimation of brain age based on features from the whole brain, we also performed brain age estimation of regional subsets of features (11, 13). We used the lobe parcellation labels from Freesurfer (21) to identify features that overlapped with a given lobe and performed similar machine learning procedures sets as described for the whole brain using occipital, frontal, temporal, cingulate, insula, and subcortical/cerebellar features alone, respectively.

### Statistical Analyses

We used R (R Core Team, Vienna, 2018) for statistical analyses. All LME models accounted for age, age^2^, sex, and scanner (30). We estimated annual change in BAG by dividing the total change in BAG by the relevant time interval. We utilized the longest time interval between time points and excluded MS patients lacking longitudinal data (*n* = 8). A score of 0 indicates that the rate of brain aging corresponds to chronological aging, and positive and negative values correspond to accelerated and decelerated brain aging compared to chronological aging, respectively. For each brain region we tested the relative rate of brain aging on a group level by performing one-sample *t*-tests on BAG with 0 as test value. We estimated the annual global brain atrophy by comparing estimated total brain volume from the Freesurfer output (BrainSegVolNotVent) between time points. Based on Freesurfer volumetric output, we also compared the volumetric and normalized measurements (divided by estimated total intracranial volume) between MS patients and HC (Supplementary Table 2).

To assess reliability of brain age across time we computed the intraclass correlation coefficient (ICC) using the R package “irr” (https://CRAN.R-project.org/package=irr). Figures were made using “ggplot2” (31) and “cowplot” (https://CRAN.R-project.org/package=cowplot) in R. To control for multiple testing we adjusted the *p*-values using false discovery rate (FDR) (32) procedures implemented in the R package “p.adjust” (http://stat.ethz.ch/R-manual/R-devel/library/stats/html/p.adjust.html). The LME models were performed using the R package “nlme” (https://CRAN.R-project.org/package=nlme).

## Results

### Participant Demographics and Characteristics

Table 1 summarizes the demographic and clinical characteristics of all MS patients. Key demographic variables regarding HC are summarized in Supplementary Table 2. The majority of the MS patients were women (71%), 96% had relapsing-remitting MS and mean age at inclusion was 34.8 years (±7.2). On average they were examined 1.2, 2.2, and 5.5 years after diagnosis. Most patients used first line treatment; 65, 48, and 37% at time point 1, 2, and 3, respectively. Second line treatments were used by 13, 23, and 32% of the MS patients at time point 1, 2, and 3, respectively. At time point 2 and 3, 53 and 44% of the patients were categorized as having NEDA (No Evidence of Disease Activity)−3 (no clinical progression, no new lesions observable in MRI and no new attacks). At time point 2, 43% of the patients with EDA (Evidence of Disease Activity) had changed their disease modifying treatment (DMT). At time point 3, 77% of the patients with EDA had changed their DMT.

**Table 1:** Demographic and clinical characteristics of the multiple sclerosis patients.

	**Time point 1**	**Time point 2**	**Time point 3**
**(a) Demographic characteristics**	*n* = 76	*n* = 75	*n* = 62
Female (%)	54 (71)	54 (72)	44 (71)
Age, mean years (SD)	34.8 (7.2)	35.8 (7.2)	40.0 (7.3)
≥15 years education (%)	53 (70)	NA	50 (81)
Disease duration, mean months (SD)	71.7 (63.0)	79.7 (57.1)	125.1 (60.2)
Age at first symptom, mean years (SD)	29.3 (6.7)		
Months since MS diagnosis, mean (SD)	14.0 (11.8)	26.3 (11.7)	66.2 (13.3)
Positive OCB status (%)	69 (91)		
*Disease modifying treatment*			
None (%)	17 (22)	22 (29)	19 (31)
First line (%)	49 (65)	36 (48)	23 (37)
Second line (%)	10 (13)	17 (23)	20 (32)
**(b) Clinical evaluation**			
*Multiple sclerosis classification*			
RRMS (%)	73 (96)	72 (96)	60 (95)
PPMS (%)	2 (3)	2 (3)	1 (2)
SPMS (%)	1 (1)	1 (1)	2 (3)
*Neurological disability*			
EDSS, median (SD, range)	2.0 (0.9, 0-6)	2.0 (0.9, 0-4)	2.0 (1.3, 0-6)
MSSS (SD)	4.9 (1.9)	4.5 (2.0)	2.6 (1.8)
Number of total attacks, mean (SD)	1.8 (1.0)	2.0 (1.0)	2.6 (1.3)
*Nine hole peg test*			
Dominant hand, mean seconds (SD)	20.0 (3.1)	NA	20.6 (8.4)
Non-dominant hand, mean seconds (SD)	20.8 (2.8)	NA	21.1 (5.9)
Timed 25 feet walk test, mean seconds (SD)	4.0 (0.7)	3.9 (0.8)	4.0 (1.1)
**(c) NEDA assessment**			
NEDA-3 (%)		40 (53)	27 (44)
NEDA-4 (%)		17 (30)	18 (32)

*OCB, oligoclonal bands; RRMS, relapsing-remitting multiple sclerosis; PPMS, primary progressive multiple sclerosis; SPMS, secondary progressive multiple sclerosis; EDSS, expanded disability status scale; MSSS, multiple sclerosis severity scale; NEDA, no evidence of disease activity*.

### Cross-Sectional Case-Control Analyses (3 T)

**Figure 1:**
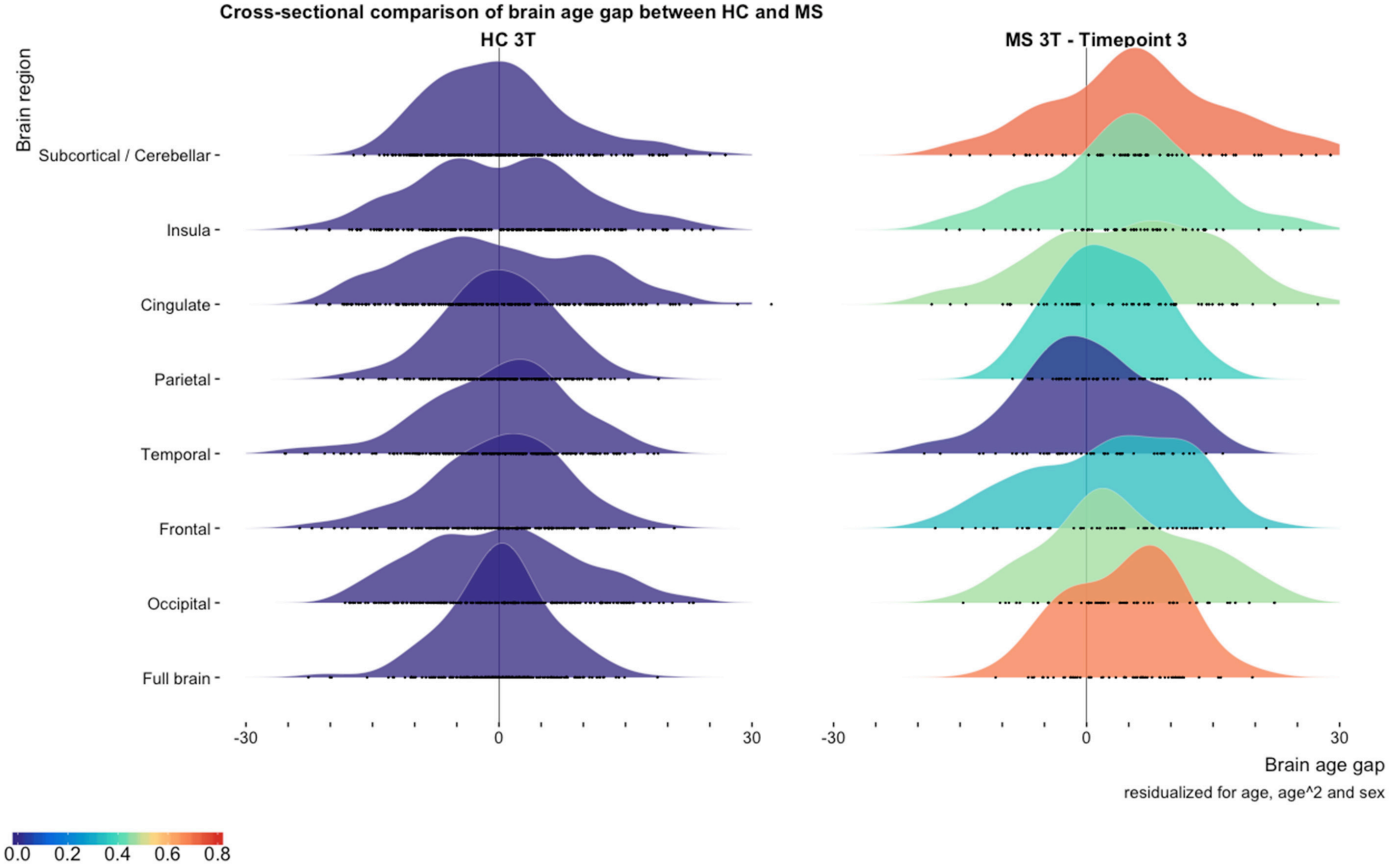
Cross-sectional comparison of brain age gap between multiple sclerosis patients and healthy controls. The distribution of brain age gaps across brain regions based on the cross-sectional 3 T MRI data from matched HC and multiple sclerosis patients at time point 3. We found increased brain age gaps for all brain regions except from the temporal brain region. Brain age gaps are residualized for age, age^2^, and sex. Cohen's D effect sizes for the brain age gap between HC and multiple sclerosis patients are depicted using the color bar. All BAG estimates are depicted as black circles on the x-axes.

At time point 3 (3T data) we found significantly higher BAG for the MS group compared to matched HC for all brain regions except the temporal region (Figure 1; Supplementary Table 4). The most prominent differences in BAG were 4.4 years for global BAG (Cohen's D = 0.69) and 6.2 years for subcortical and cerebellar brain regions (Cohen's D = 0.72).

At time point 3, 58 MS patients underwent one MRI scanning in the 1.5 T and one in the 3 T scanner with 2 days apart. Whereas, absolute estimates of brain age varied between scanners for all brain regions except insula (BAG scanner difference −6.08 to 10.60 years, see Supplementary Table 3; Supplementary Figures 4), brain age estimates from the two scanners were highly correlated for global BAG and all brain regions (*r* = 0.67–0.86, *p* < 0.001), supporting the reproducibility.

Volumetric data showed no significant differences in measures of whole brain, gray matter and white matter. When using normalized measurements (divided by estimated total intracranial volume), we found significant differences between normalized whole brain (Cohen's D = 0.45) and gray matter (Cohen's D = 0.46) volumes (Supplementary Table 2).

### Longitudinal MS Sample (1.5 T)

The correlations between chronological age and global brain age were *r* = 0.71 for time point 1, *r* = 0.70 for time point 2, and *r* = 0.69 for time point 3. After adjusting for scanner effects mean global BAG was 2.8 (±9.0) for time point 1, 3.3 (±9.4) for time point 2, and 4.6 (±9.8) for time point 3 in the longitudinal MS sample (Supplementary Figures 5A,B). Some patients exhibited reduced estimates of brain age over time, likely partly explained by an effect of MRI noise characteristics (subject motion, MRI artifacts or any other technical changes between acquisitions), while in the same period the biological changes were negligible.

**Figure 2:**
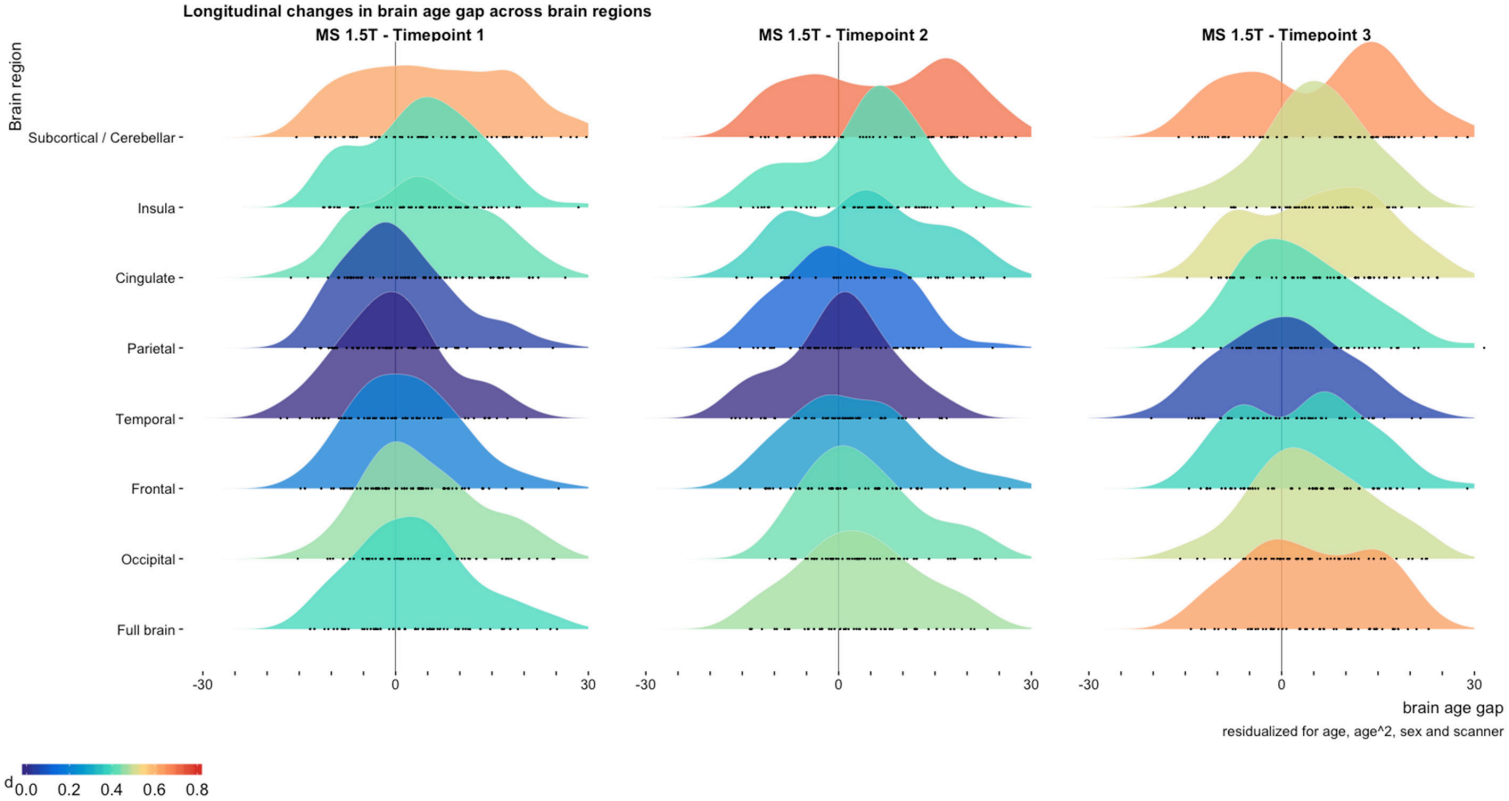
Longitudinal changes in brain age gap across brain regions. The distribution of brain age gaps across brain regions based on the longitudinal 1.5 T MRI sample. Brain age gaps from the MS sample are compared with the cross-sectional 3 T HC sample and residualized for age, age^2^, sex, and scanner. The full brain estimates showed a significant accelerated rate of brain aging compared to chronological aging [annual increase in brain age gap 0.41 (*p* = 0.008)]. Cohen's D effect sizes for the brain age gap between MS and HC are depicted using the color bar. All BAG estimates are depicted as black circles on the x-axes.

We found a significant annual increase in global BAG of 0.41 (SE = 0.15) years (*p* = 0.008) in patients with MS (Figure 2; Supplementary Tables 6, 7). No regional measures showed significantly decreasing or increasing BAG at the group level (Supplementary Tables 7, 8). Our dataset included a low number of other MS phenotypes than RRMS, and we did not find significant correlations between brain atrophy rates or annual change in global BAG among these.

We found no significant difference in BAG between the raw and the lesion filled MRI scans, and the BAG scores from the two versions were highly correlated BAG (*r* = 0.98). Data processed with the longitudinal stream in FreeSurfer had significantly lower BAG than the cross-sectionally processed MRI scans (mean difference in BAG 4.9 years, *p* < 0.001) and lesion filled MRI scans (difference in BAG 5.1 years, *p* ≤ 0.001) (Supplementary Figures 6, 7; Supplementary Tables 4, 5).

ICCs for all brain regions across all time points varied from 0.79 to 0.94 for residualized BAG and 0.78–0.95 for predicted age. Cerebellar and subcortical brain regions showed highest reliability with an ICC of 0.94 for BAG and 0.95 for predicted age (Supplementary Table 9).

Mean annualized estimated change in global brain volume from all three time points. from Freesurfer was −0.30% (SD = 0.53%). ICC for global brain volume was 0.97–0.99. Mean annualized change in WMLL was 504 mm^3^ (±28 mm^3^). ICC for WMLL at time point three was 0.93–0.99.

### Associations Between Global Brain Age and Clinical Outcomes

Table 2 (BAG) and Table 3 (annual rate of brain aging) show summary statistics from the multiple regressions testing for associations with demographic, clinical, and MRI variables in the longitudinal MS group (Supplementary Tables 10, 11). After accounting for multiple testing, significant associations were found between BAG and WMLL (Cohen's D = −1.23, *p* = 3.0 × 10^−4^) and global brain atrophy (Cohen's D = −0.07, *p* = 0.01), respectively, indicating higher BAG at baseline with higher WMLL at time point three and increased brain atrophy over time. Further, changes in BAG over time was significantly associated with brain atrophy over time (Cohen's D = 0.86, *p* = 4.3 × 10^−15^) and change in WMLL (Cohen's D = 0.55, *p* = 0.015), indicating higher rates of brain aging in patients with higher levels of brain atrophy and more progressive changes in WMLL. WMLL also showed a significant correlation with BAG for cerebellar and subcortical regions (Cohen's D = −1.23, *p* = 3.2 × 10^−3^).

**Table 2:** Pearson's correlations between brain age gap and relevant clinical and MRI variables.

	**Fullbrain**		**Frontal**		**Parietal**		**Cereb. / Subcort**.	
**Clinical variables**	**cor**.	***p***	**cor**.	***p***	**cor**.	***p***	**cor**.	***p***
9HPT Non-dominant	**0.36**	**5.8** **×** **10**^**−3**^	0.03	0.80	0.16	0.22	**0.28**	**0.030**
Change in 9HPT Non-dominant	**0.28**	**0.035**	0.05	0.68	0.14	0.31	0.21	0.12
DMT Level	0.01	0.93	0.03	0.80	−0.05	0.70	**0.26**	**0.046**
Gender	**−0.28**	**0.031**	0.05	0.68	−0.18	0.17	−0.04	0.78
**MRI variables**	**cor**.	***p***	**cor**.	***p***	**cor**.	***p***	**cor**.	***p***
WMLL	**0.46**	**3.0×10**^**−4**^	0.19	0.16	0.24	0.07	**0.38**	**3.2×10**^**−3**^
Change in WMLL	**0.30**	**0.022**	0.12	0.34	0.20	0.13	**0.34**	**9.6** **×** **10**^**−3**^
Brain volume	−0.25	0.06	****−**0.43**	**8.8×10**^**−4**^	****−**0.35**	**7.3×10**^**−3**^	−0.24	0.07
Brain atrophy	****−**0.33**	**0.011**	****−**0.31**	**0.017**	****−**0.37**	**4.7×10**^**−3**^	−0.13	0.32
ICV	−0.01	0.94	**−0.29**	**0.027**	−0.20	0.13	−0.02	0.87

*Significant associations are highlighted with bold (p < 0.05). Associations which were still significant after adjusting for false discovery rate are underlined. Cereb., cerebellar; Subcort., subcortical; 9HPT, nine hole peg test; Cor., correlation; DMT, disease-modifying therapies; WMLL, white matter lesion load; ICV, intracranial volume*.

**Table 3:** Pearson's correlations between annual rate of brain aging and relevant clinical and MRI variables on time point 3.

	**Fullbrain**		**Frontal**		**Parietal**		**Cereb. / Subcort**.	
**Clinical variables**	**cor**.	***p***	**cor**.	***p***	**cor**.	***p***	**cor**.	***p***
EDSS	0.09	0.49	−0.01	0.95	−0.15	0.25	0.22	0.08
Change in EDSS	0.16	0.23	0.09	0.50	−0.03	0.83	**0.29**	**0.026**
MSSS	−0.03	0.84	−0.09	0.47	−0.21	0.11	0.17	0.20
Change in MSSS	0.17	0.21	0.10	0.46	0.05	0.68	**0.36**	**5.1** **×** **10**^**−3**^
9HPT Non-dominant	**0.29**	**0.028**	0.15	0.27	0.01	0.92	**0.30**	**0.021**
Change in 9HPT Non-dominant	**0.31**	**0.017**	0.20	0.14	0.08	0.53	**0.32**	**0.014**
DMT Level	**−0.28**	**0.031**	−0.22	0.09	−0.17	0.21	−0.08	0.54
**MRI variables**	**cor**.	***p***	**cor**.	***p***	**cor**.	***p***	**cor**.	***p***
WMLL	**0.29**	**0.026**	0.21	0.11	0.19	0.16	0.01	0.96
Change in WMLL	**0.30**	**0.015**	0.19	0.12	**0.35**	**4.3×10**^**−3**^	0.00	0.98
Brain volume	−0.01	0.93	−0.08	0.54	−0.03	0.83	0.10	0.44
Brain atrophy	****−**0.79**	**4.3×10**^**−15**^	****−**0.79**	**1.6×10**^**−15**^	****−**0.72**	**1.1×10**^**−11**^	−0.07	0.57

*Significant associations are highlighted with bold (p < 0.05). Associations which were still significant after adjusting for false discovery rate are underlined. Cereb., cerebellar; Subcort., subcortical; Cor., correlation; EDSS, expanded disability status scale; MSSS, multiple sclerosis severity score; 9HPT, nine hole peg test; DMT, disease-modifying therapies; WMLL, white matter lesion load*.

## Discussion

Using cross-sectional and longitudinal MRI data as basis for brain age estimation based on machine learning, we tested the hypotheses that patients with MS on average show higher brain age than healthy controls, and that the rate of brain aging is associated with clinical trajectories. Cross-sectional analysis revealed higher brain age gap in patients with MS compared to healthy controls, and longitudinal analysis showed increased rates of brain aging in patients with higher rates of brain atrophy and increasing WMLL.

MS patients had on average 4.4 years higher BAG compared to HC (Cohen's D = 0.68), in line with preliminary findings in a partly overlapping cross-sectional sample (11). To our knowledge, other studies comparable to ours are not yet available, and further studies are warranted. Global brain age differences may disguise relevant regional effects. Indeed, for subcortical and cerebellar brain regions we found a higher BAG in MS compared with HCs (BAG 6.2 years, Cohen's D = 0.72), which was already evident at time point 1 (BAG 5.7 years, Cohen's D = 0.63). The regional variability may reflect differential affinity of MS pathology across the brain, which is also supported by lesion probability maps in MS (2, 33).

In our longitudinal patient sample, the average annual rate of brain aging for global BAG exceeded that of chronological aging by 0.41 years per year (*p* = 0.008). Although further studies are needed, this apparent accelerated aging of the brain may partly be explained by chronic inflammatory processes that drive neurodegeneration in MS (1).

As expected, we found relatively robust associations between brain atrophy and brain aging (*r* = 0.79, *p* = 4.3 × 10^−15^). Of notice, regional brain aging and BAG is sensitive to subtle brain changes that may not necessarily be picked up in the global brain atrophy measures. Indeed, the associations between change in WMLLs and annual rate of brain aging were significant for occipital, temporal, and parietal brain regions in addition to the global estimate (Supplementary Table 10). For BAG we did indeed only see significant associations with brain volume and BAG for occipital, frontal, parietal, and cingulate regions (Supplementary Table 11). This shows that regional brain age estimation may capture regional specificity of MS pathology (10–12, 33).

Multiple regression analyses revealed only nominally significant (*p* < 0.05, uncorrected) associations between some clinical, cognitive, and imaging variables and BAG as well as brain aging for specific brain regions. However, these associations did not survive correction for multiple testing, and further studies are needed to assess the robustness of these observations. A previous study in healthy individuals reported significant associations between BAG and performance on specific cognitive tests, including spatial Stroop and symbol coding, with poorer performance in individuals with an over-estimated age (13). Preliminary results from a partly overlapping cross-sectional sample revealed a significant association between BAG and Expanded Disability Status Scale (Fisher *z* = 0.23) (11), indicating that patients with higher clinical disease burden have older appearing brains. Further studies are needed to test the generalizability and robustness of these findings, both in clinical and healthy samples.

Brain age estimation is a useful framework that allows us to leverage large scale brain imaging databases for training robust machine learning models and apply automated prediction on the individual level. Further, whereas the approach builds on the vast amount of previous atrophy and lesion research, it contributes beyond that by downsampling a lot of information from the entire brain into a single holistic score in an automated fashion. As an example, in our data, we found no association between brain atrophy and change in Multiple Sclerosis Severity Scale (MSSS) (*r* = 0.03, *p* = 0.80), yet our brain age estimation approach revealed associations with change in MSSS for brain aging of the cerebellar & subcortical regions (*r* = 0.36, *p* = 5.1 × 10^−3^, not significant after correcting for multiple testing) (Supplementary Table 12).

Some limitations should be considered when interpreting the results. First, although the cross-sectional case-control comparison and the within-patient longitudinal analysis jointly suggest accelerated brain aging in patients with MS, a longitudinal sample of HCs would have enabled us to directly compare the rate of brain aging between patients and controls. Next, the current brain age model was exclusively based on gross morphometric features, and extending the range of brain imaging features, including indices of white matter microstructural properties and myelin integrity, may increase sensitivity to clinical trajectories in MS. When analyzing clinical associations with estimates of brain age gap we include clinical tests which relies heavily on the spine, although morphometric data from the spine are not included in our brain age estimation model. Finally, although prospective data is a substantial strength of our study, our design does not allow for causal inference (e.g., related to treatment status). Data from analyses of brain age compared to disease modifying treatments are provided in Supplementary Figure 8, and Supplementary Tables 10, 11, 13. Our current brain age estimation model aimed at identifying deviations from healthy aging trajectories, future studies could potentially benefit from establishing unique models based on disease specific training sets.

In conclusion, using advanced cross-sectional imaging data and machine learning we report higher brain age in patients with MS compared to healthy controls. Longitudinal analysis suggested accelerated brain aging in MS patients with higher levels of brain atrophy and longitudinal progression of changes in WMLL. Brain age estimation is a framework that allows us to downsample the complex brain imaging features into a single individual “score” using automated machine learning, enabling us to gain new insights into the complex brain structure. Jointly, these results corroborate that brain age estimation is a promising and intuitive tool with potential to establish a comprehensive measure of brain health which may guide a personalized treatment approach in MS.

## Ethics Statement

This study was carried out in accordance with the recommendations of the Regional Committee for Medical and Health Research Ethics with written informed consent from all subjects. All subjects gave written informed consent in accordance with the Declaration of Helsinki. The protocol was approved by the South East Regional Committee for Medical and Health Research Ethics.

## Author’s Note

A preprint of this manuscript was published at bioRxiv (https://www.biorxiv.org/content/10.1101/440412v1) on October 10th 28 (34).

## Author Contributions

EH, TK, GN, HH, and LW contributed to the conception and design of the study. EH, TK, GN, KK, GR, HH, and LW contributed to the acquisition and analysis of data. EH, TK, GN, KK, HH, and LW drafted the text and figures. All authors contributed to the review and editing.

### Conflict of Interest Statement

EH has received honoraria for lecturing from Merck and Sanofi-Genzyme. MB has received honoraria for lecturing from Novartis and Biogen Idec. OA has received honoraria for lecturing from Lundbeck. HH has received travel support, honoraria for advice or lecturing from Biogen Idec, Sanofi-Genzyme, Merck, Novartis, Roche, and Teva and an unrestricted research grant from Novartis. The remaining authors declare that the research was conducted in the absence of any commercial or financial relationships that could be construed as a potential conflict of interest.

## Abbreviations

BAG: Brain Age Gap
Cereb.: Cerebellar
DMT: Disease Modifying Treatment
EDA: Evidence of Disease Activity
HC: Healthy Controls
ICC: Intraclass Correlation Coefficient
MS: Multiple Sclerosis
MSSS: Multiple Sclerosis Severity Scale
NEDA: No Evidence of Disease Activity
Subcort.: Subcortical
WMLL: White Matter Lesion Load.
