# Supplementary material for: Cross-Sectional and Longitudinal MRI Brain Scans Reveal Accelerated Brain Aging in Multiple Sclerosis
Source: Front Neurol. 2019 Apr 30;10:450. doi: 10.3389/fneur.2019.00450 (PMC6503038; doi:10.3389/fneur.2019.00450)
Supplement: Supplementary file 1 [file Data_Sheet_1.PDF]

SUPPLEMENTARY MATERIAL

Manual editing of the automatically acquired FreeSurfer segmentation was performed after standard quality control of all the MRI brain scans (Kaufmann *et al.*, 2018). We utilized the same MRI processing pipeline as described in detail in the preprint by Kaufmann et al (Kaufmann *et al.*, 2018).

Brain aging in association with DMT

To assess brain aging within groups stratified by DMT, we used the coefficients from linear models with the individual slope in BAG as dependent variable and age, age^2, sex and DMT group as independent variables.

In the longitudinal data, One Sample t-test revealed a significant increase in BAG in DMT group 0 (0.92 (±0.82), $p = 5.4x10^{-4}$), and no significant changes in DMT groups 1 (0.13 (±1.3), p = 0.63), and 2 (0.35 (±1.3), p = 0.26). However, linear models and a LME analysis revealed no significant group differences in the rate of brain aging (f-value = 2.47, p = 0.09) (Supplementary Fig. 7).

The significant increase in BAG in the patients receiving no treatment warrants further investigations but is of clinical interest. However, the lack of randomization and group differences in the subgroups calls for caution in the interpretations of this relatively small dataset.

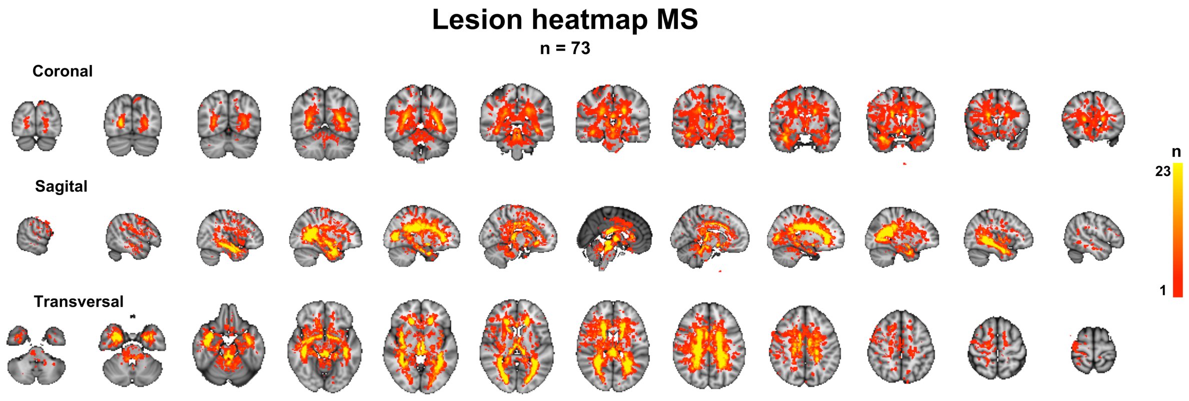

Supplementary figure 1. Heat map showing the distribution of MS lesions in the brain. Utilizing automatically generated lesion masks from Cascade (Damangir *et al.*, 2012), we plotted all normalized MS lesions from time point 1 on the standardized MNI-152–template to visualize the distribution of the MS lesions in the brain. Depicted are slices in the coronal,

sagital and transversal plane evenly distributed. Increasing yellow colour represents higher lesion count, as depicted in the colour bar.

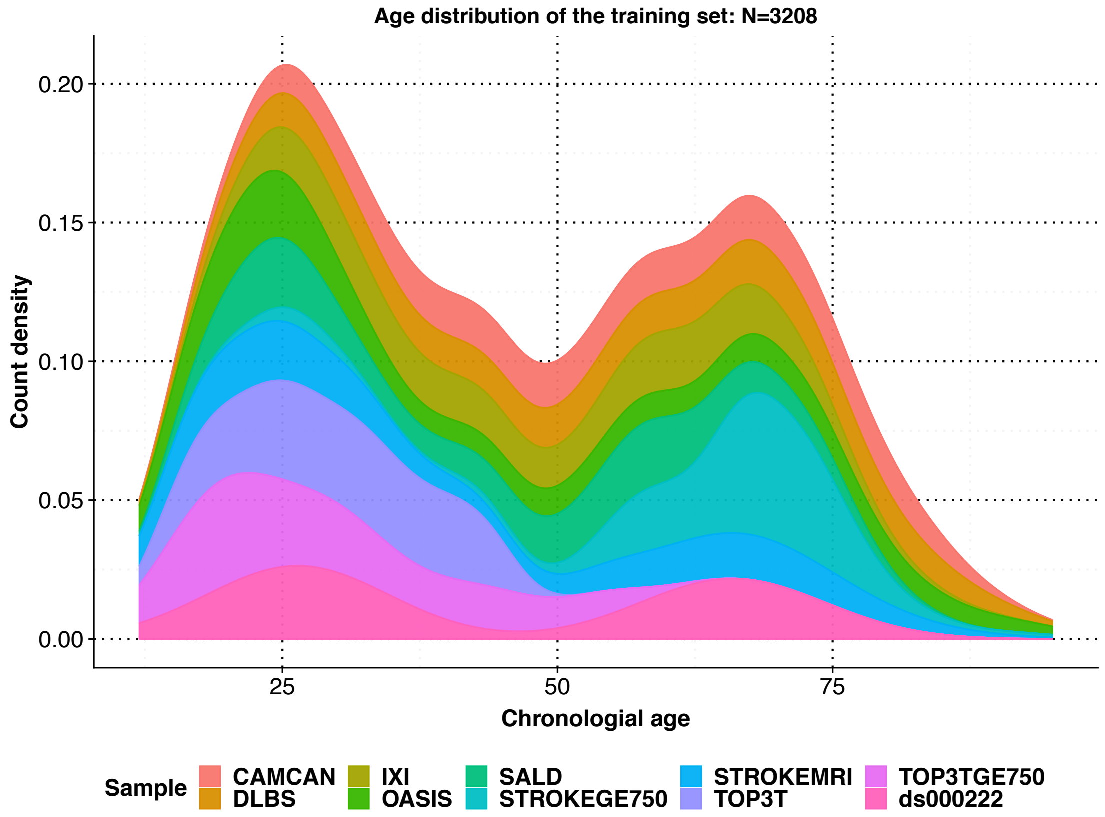

Supplementary figure 2. Overview age distribution of the training set. Histogram showing the age distribution of the training set, including the age distributions of the different cohorts using different colours. Information concerning the different sample cohorts (CAMCAN, DLBS, IXI, OASIS, SALD, STROKEGE750, STROKEMRI, TOP3T, TOP3TGE750 and ds000222) contributing to the training set are available in the article by Kaufman et al (Kaufmann *et al.*, 2018).

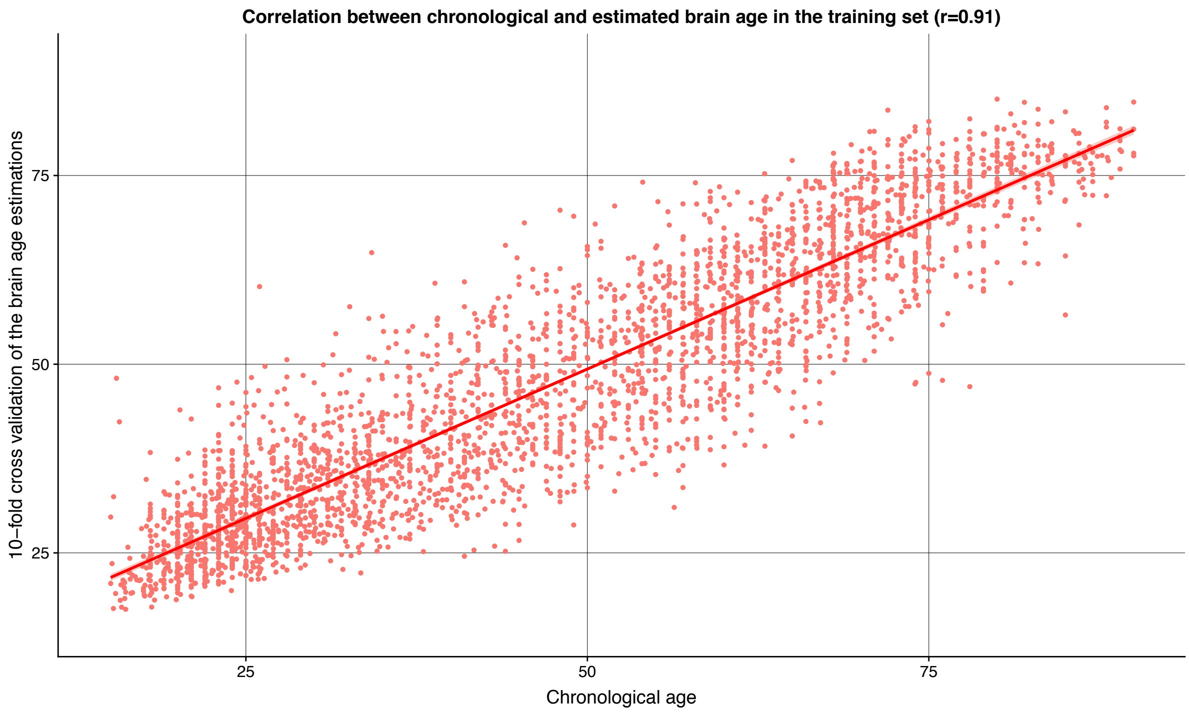

Supplementary figure 3. Result of the brain age estimates from the training set. Correlations between brain age estimations and age after performing a 10-fold cross validation in the training set (r = 0.91). We found a shift in the estimations <50 and >50 years of age, with increasing overestimation of age <50 years and increasing underestimation of age >50 years, which is a known effect when utilizing a machine learning model to estimate brain age (Cole and Franke, 2017; Kaufmann *et al.*, 2018).

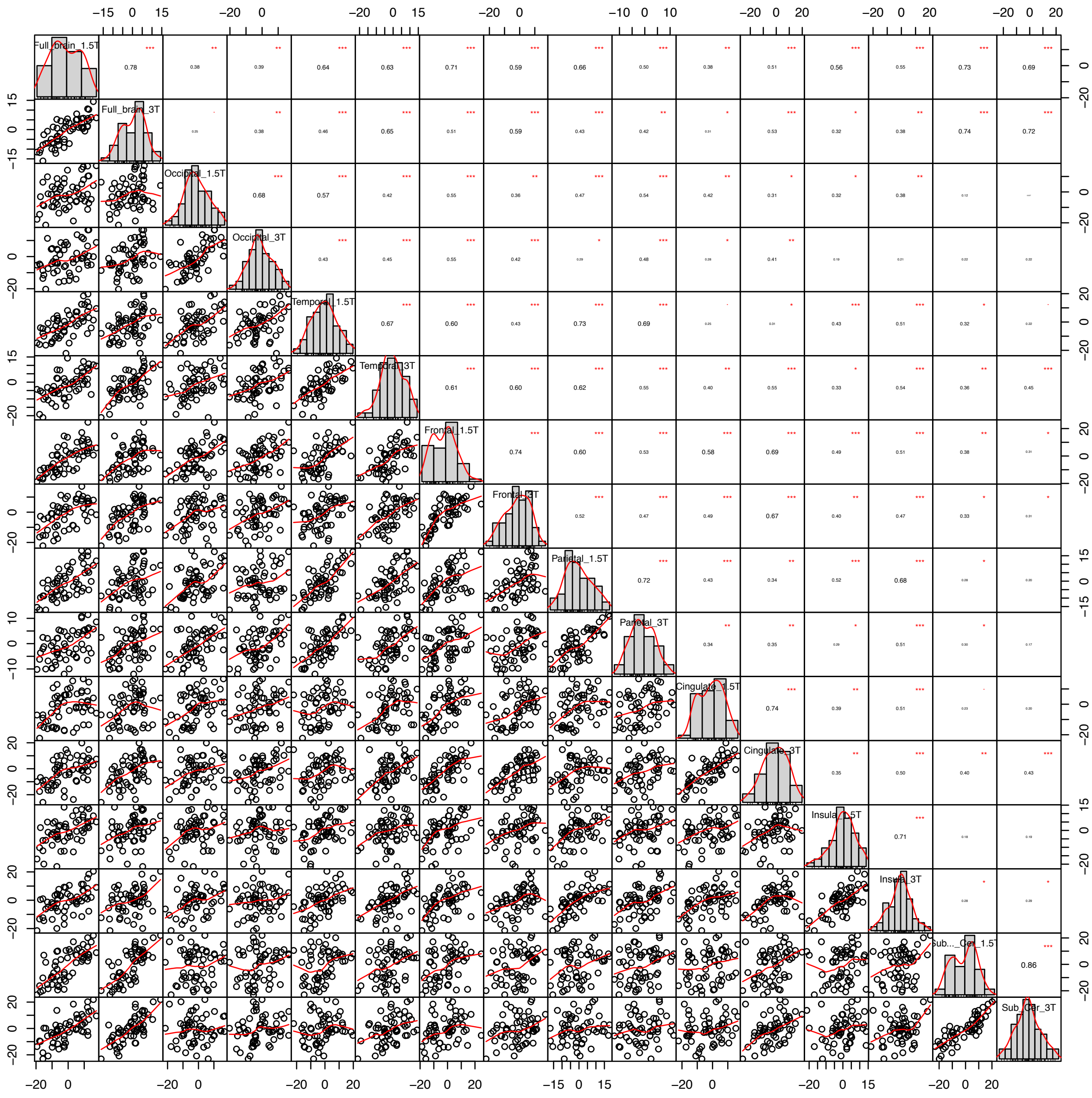

Supplementary figure 4. Correlation matrix between the two MRI scanners. A chart of the correlation matrix for the comparison of the BAG estimates for global and all brain regions from the 1.5 T and 3 T MRI scanner (n = 58). The distribution of each variable is shown on the diagonal. On the bottom of the diagonal we see the bivariate scatter plots displayed with a fitted line. On the top of the diagonal we show the value of the correlation plus the significance level as stars and symbols (p-values; "***" = 0-0.001, "**" = 0.001-0.01, "*" = 0.01-0.05, "." = 0.05-0.1, " " = 0.1-1). The chart is made using the "PerformanceAnalytics" package in R.

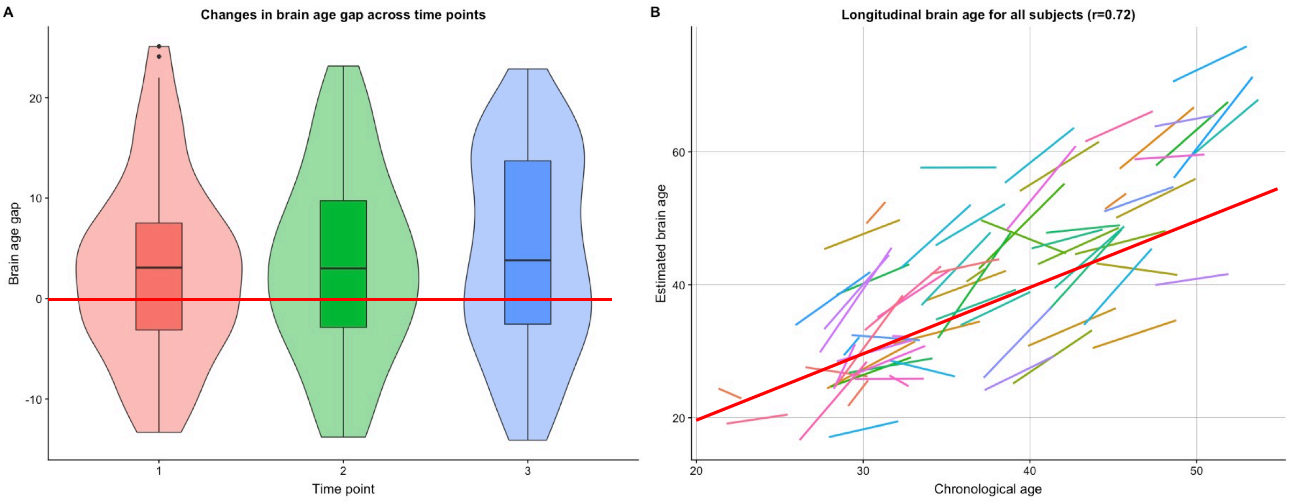

Supplementary figure 5. Visualization of brain age in the longitudinal MS cohort. A) Difference between chronological and predicted age (brain age gap) are shown for all three time points separately. After adjusting for scanner effects mean brain age gap was 2.8 (±9.0) for time point 1, 3.3 (±9.4) for time point 2 and 4.6 (±9.8) for time point 3 in the longitudinal sample. The distributions of the brain age estimates are visualized using box and violin plots. The red line illustrates the expected chronological aging as BAG would then expected to be 0 at all timepoints. **B**) Multiple sclerosis subjects are depicted with linear brain age slopes using linear regression models to visualize individualised estimations of brain aging. Only participants with more than one MRI scan are included (n = 68). Mean annual increase in global BAG was 0.41(±1.23) years (p = 0.008) in patients with multiple sclerosis. The red line illustrates the expected chronological aging.

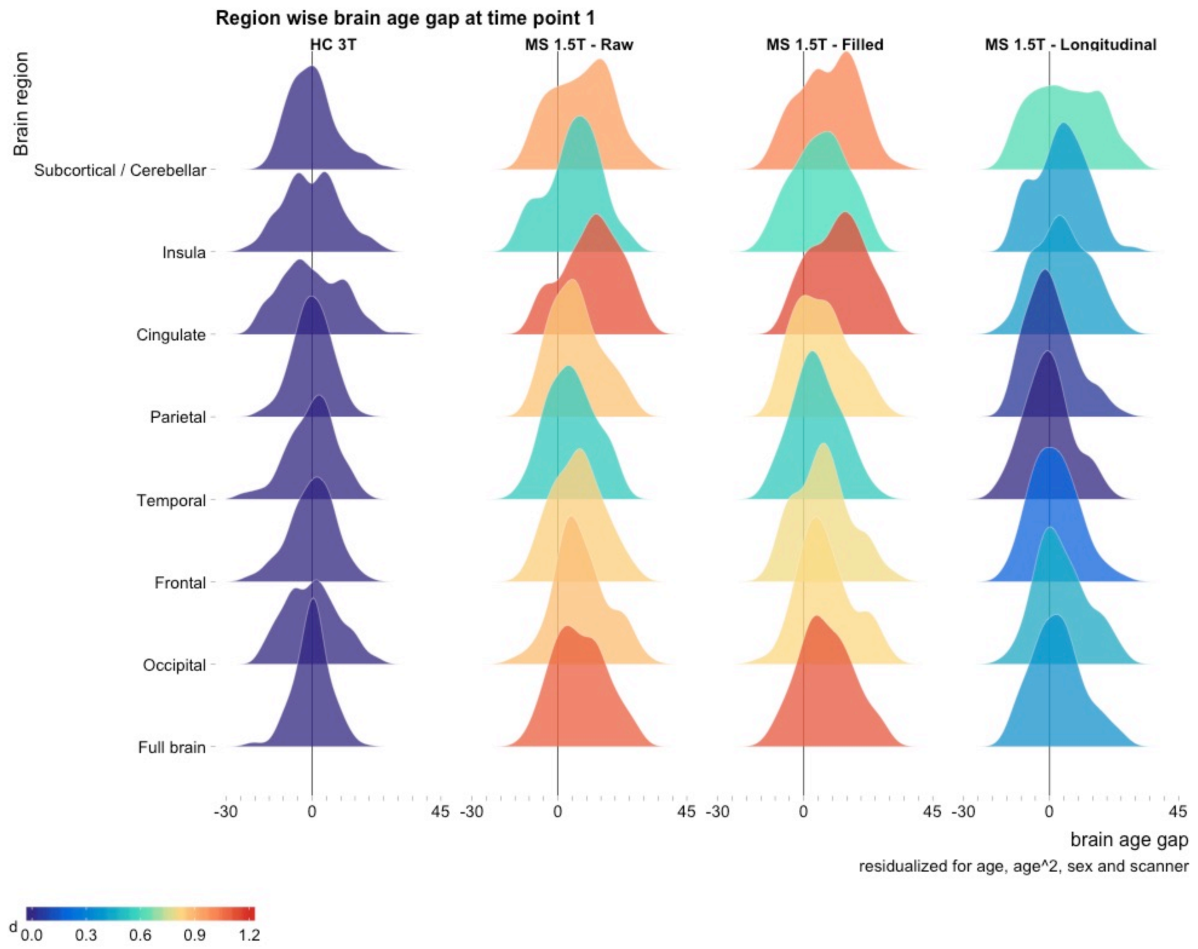

Supplementary figure 6. Overview of all brain age estimations from time point 1. The distribution of brain age gaps across brain regions based on MRI data from time point 1 (raw, lesion filling and longitudinal stream). Resulting brain age gaps from the three different processing steps are compared with the HC sample after we controlled for age, age^2, sex and scanner. Shown are the resulting brain age estimations from the raw images, raw images including lesion filling and images after being processed through the FreeSurfer longitudinal stream. All three processing steps were highly correlated when compared group wise using the resulting predicted brain age (r>0.95). Cohen's D effect sizes for the brain age gap between MS and HC are depicted using the colour bar.

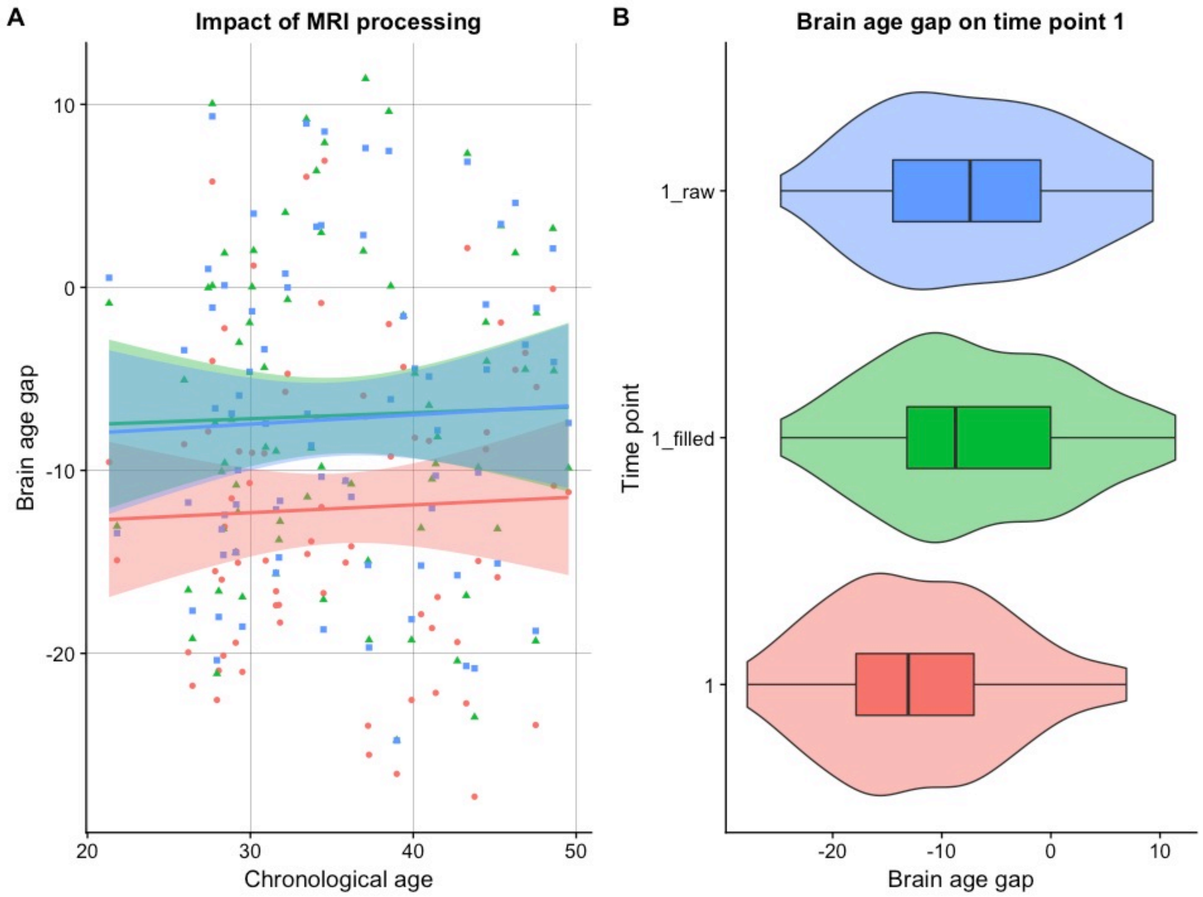

Supplementary figure 7. The effects on brain age gap before correcting for scanner effects by different processing steps of the MRI data from time point 1. A, The associations between chronological age and brain age gap are plotted. Depicted in red are the resulting brain age estimations from the FreeSurfer longitudinal stream, in green the brain age estimations from the MRI scans after lesion filling and in blue are the brain age estimations from the raw images. **B,** Plots visualizing the same as in **A,** using box plots to show the distribution of the brain age estimates. Brain age gap for each of the groups are -2.4, 2.6 and 2.4 for the red, green and blue groups respectively. All three processing steps were highly correlated group wise using the resulting predicted brain age (r>0.95). Brain age gaps were residualized for age, age^2 and sex to minimize confounding effects of the variables.

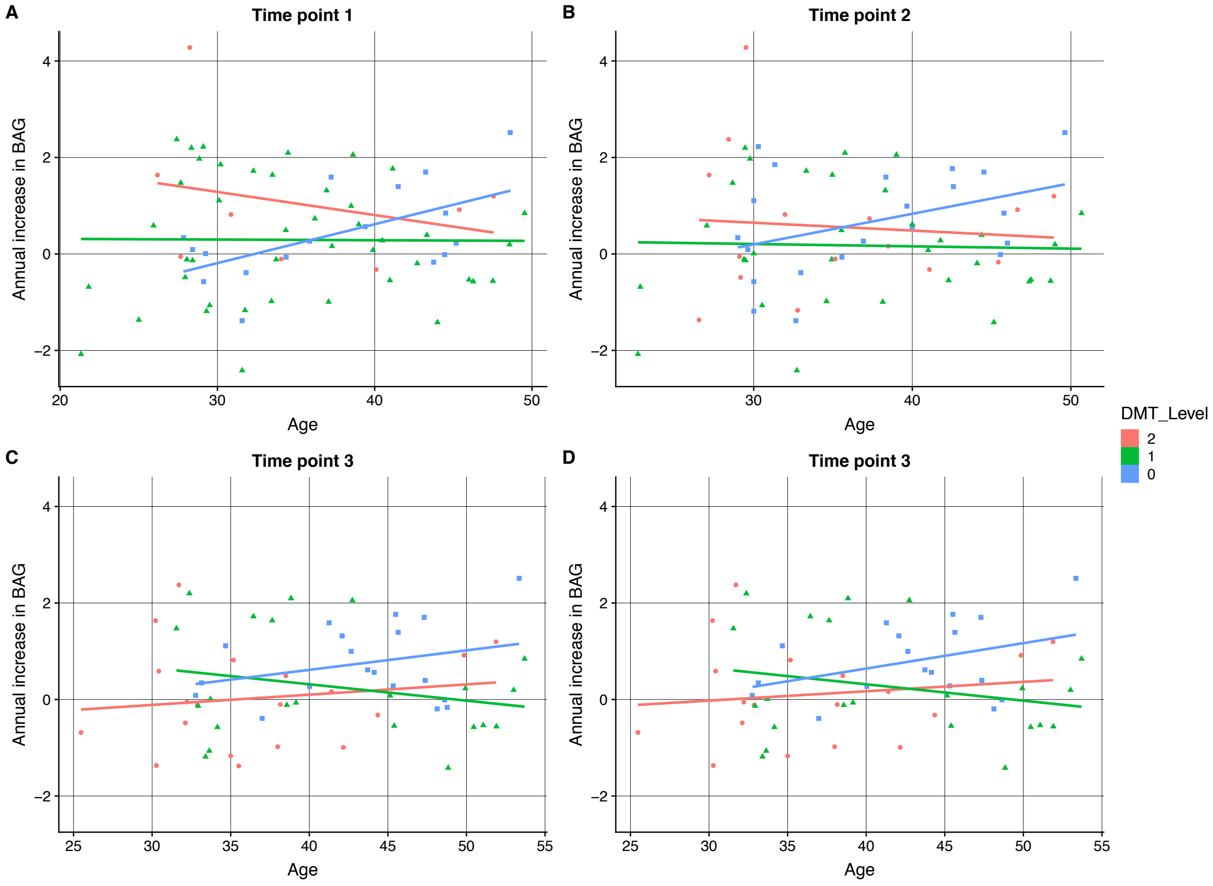

Supplementary figure 8. Associations between annualized brain aging rate and chronological age in the MS sample. Subjects are marked as coloured symbols, depending on their DMT level, and grouped together to visualize possibly the trajectories for each group independently. All data are derived from global BAG estimates, residualized for age, age^2 and sex. Analyses showed no significant associations between DMT treatment and rate of brain aging. **A,** DMT level at time point 1. **B,** DMT level at time point 2. **C.** DMT level at time point 3. **D,** DMT level at time point 3, also taking into account retrospective DMT switches, defined into three groups.
