# Supplementary material for: Cross-Sectional and Longitudinal MRI Brain Scans Reveal Accelerated Brain Aging in Multiple Sclerosis
Source: Front Neurol. 2019 Apr 30;10:450. doi: 10.3389/fneur.2019.00450 (PMC6503038; doi:10.3389/fneur.2019.00450)
Supplement: Supplementary file 2 [file Data_Sheet_2.PDF]

Supplementary table 1. Comparison of prediction models in our data. The table compares the performance of predicted age and residualized brain age gap from xgboost and shrinkage linear models (SLM) from the full brain and all brain regions. Correlations between the variables were calculated with Pearson r and reported for each analysis.

**Age vs predicted age (xgboost)**	***MS 1.5T (n=189 scans)***	***MS 3T (n=56 scans)***	***HC 3T (n=233)***	***All (n=482 scans)***
Brain regions				
Full brain	r=0.60	r=0.50	r=0.63	r=0.56
Occipital	r=0.23	r=0.04	r=0.35	r=0.24
Temporal	r=0.46	r=0.36	r=0.46	r=0.46
Frontal	r=0.50	r=0.40	r=0.58	r=0.52
Parietal	r=0.52	r=0.49	r=0.45	r=0.49
Cingulate	r=0.33	r=0.35	r=0.39	r=0.24
Insula	r=0.41	r=0.49	r=0.41	r=0.31
Cerebellar / Subcortical	r=0.43	r=0.37	r=0.54	r=0.39
**Age vs. predicted age (slm)**				
Full brain	r=0.54	r=0.54	r=0.54	r=0.52
Occipital	r=0.23	r=0.43	r=-0.10	r=0.19
Temporal	r=0.33	r=0.18	r=0.34	r=0.33
Frontal	r=0.37	r=0.36	r=0.44	r=0.39
Parietal	r=0.26	r=0.20	r=0.37	r=0.27
Cingulate	r=0.26	r=0.06	r=0.18	r=0.22
Insula	r=0.40	r=0.42	r=0.32	r=0.32
Cerebellar / Subcortical	r=0.48	r=0.49	r=0.53	r=0.41
**Predicted age (xgboost) vs. predicted age (slm)**				
Full brain	r=0.62	r=0.67	r=0.49	r=0.57
Occipital	r=-0.05	r=-0.16	r=-0.21	r=-0.18
Temporal	r=0.54	r=0.58	r=0.57	r=0.56
Frontal	r=0.66	r=0.76	r=0.63	r=0.66
Parietal	r=0.39	r=0.42	r=0.49	r=0.43
Cingulate	r=0.40	r=0.27	r=0.44	r=0.32
Insula	r=0.57	r=0.59	r=0.65	r=0.62
Cerebellar / Subcortical	r=0.91	r=0.88	r=0.86	r=0.90
**Brain age gap (xgboost) vs. brain age gap (slm)**				
Full brain	r=0.44	r=0.55	r=0.21	r=0.37
Occipital	r=-0.12	r=-0.26	r=-0.19	r=-0.19
Temporal	r=0.44	r=0.57	r=0.49	r=0.48
Frontal	r=0.33	r=0.43	r=0.42	r=0.40
Parietal	r=0.44	r=0.55	r=0.21	r=0.37
Cingulate	r=0.34	r=0.24	r=0.42	r=0.37
Insula	r=0.52	r=0.50	r=0.59	r=0.56
Cerebellar / Subcortical	r=0.89	r=0.87	r=0.80	r=0.87

Supplementary table 2. Age and sex distribution of the matched MS and HC samples at time point 3, including the predicted brain age and brain age gaps for global BAG estimates. We have included basic Freesurfer volumetric data and performed two sample students t-tests to test for differences between volumetric measures for both 1.5 T and 3 T MS pasients and for 3T MS patients and 3T healthy controls. Significant differences between the groups are marked in bold. Normalised measurements of FreeSurfer are given as unit-less tissue fractions in %.

Cross validation of Brain Age Prediction	**Time point 3 1,5T (n=58)**	*t-test MS 1,5T vs MS 3T*	**Time point 3 3T (n=58)**	*t-test MS 3T vs HC 3T*	**Healthy controls 3T (n=235)**
Age (SD, min-max)	40.8 (7.3, 25-53)		40.7 (7.3, 25-53)		40.8 (7.6, 26-53)
Female (%)	72 %		72 %		72 %
Predicted brain age (SD)	45.9 (11.3)		55.6 (7.8)		51.2 (8.2)
Predicted brain age gap (SD)	5.2 (9.3)		14.9 (7.0)		10.4 (7.2)
FreeSurfer volumetric data					
Whole brain, median ml (SD)	1115.1 (101.0)	p=0.75	1112.1 (101.1)	p=0.23	1120.0 (978.9)
Grey matter, median ml (SD)	617.7 (51.9)	p=0.49	630.7 (52.3)	p=0.08	646.3 (50.7)
White matter, median ml (SD)	526.0 (57.1)	p=0.22	510.6 (56.2)	p=0.53	503.2 (55.1)
Normalised whole brain, (SD)	71.0 (2.6)	**$p=1.1 \times 10^{-4}$**	72.7 (2.7)	**$p=3.1 \times 10^{-3}$**	74.0 (2.6)
Normalised grey matter, (SD)	40.0 (2.0)	**$p=1.0 \times 10^{-5}$**	41.8 (2.1)	**$p=5.0 \times 10^{-3}$**	42.3 (1.9)
Normalised white matter, (SD)	32.6 (1.7)	p=0.42	32.8 (1.5)	p=0.12	33.2 (1.8)
Cerebellum, median ml (SD)	131.6 (13.7)	p=0.14	135.0 (14.0)	p=0.18	137.7 (12.8)
Corpus callosum, median ml (SD)	3.0 (0.5)	p=0.27	3.0 (0.5)	**p=0.02**	3.1 (0.5)
Thalamus, median ml (SD)	14.3 (1.6)	p=0.84	14.3 (1.6)	**$p=1.1 \times 10^{-4}$**	15.1 (1.5)
Caudate, median ml (SD)	7.0 (1.0)	p=0.61	7.1 (1.1)	**p=0.03**	7.4 (0.9)
Putamen, median ml (SD)	10.4 (1.2)	p=0.66	10.4 (1.5)	**$p=9.8 \times 10^{-4}$**	11.0 (1.3)
Pallidum, median ml (SD)	2.8 (0.4)	p=0.95	2.8 (0.4)	**$p=3.4 \times 10^{-5}$**	3.0 (0.4)
Hippocampus, median ml (SD)	8.0 (0.8)	**p=0.03**	8.3 (0.9)	**p=0.01**	8.7 (0.8)
Amygdala, mean cm^3 (SD)	3.0 (0.4)	**p=0.01**	3.3 (0.4)	p=0.91	3.2 (0.4)

Supplementary table 3. Listed are the differences in global and regional brain age estimates and their correlation between the 1.5T and 3T MRI scanner. The data is based on time point 3 and 58 MS patients. All brain age gap estimates are residualized for age, age^2 and sex.

1.5T vs 3T scanner differences for MS patients at time point 3

Brain Region	*BAG difference 1.5T vs 3T*	*Correlation*
Full brain	9.69	0.78
Occipital	3.34	0.68
Temporal	10.20	0.67
Frontal	10.60	0.74
Parietal	9.21	0.72
Cingulate	-6.08	0.74
Insula	-0.43	0.71
Subcortical / Cerebellar	-3.54	0.86

Supplementary table 4. Differences between the MS and HC samples across all time points, including 3T at time point 3. Brain age gaps (BAG) are residualized for age, age^2, sex and scanner. Effect sizes were made using Cohen´s D (using mean, median or rank as input) estimates and the r-value from Mann-Whitney-U tests (MWU). The t and p values reported are from the Cohen´s D analyses with mean as input. The Cohen´s D effect sizes are ranked as small (0.2-<0.5), medium (0,5-<0.8) and large (>0.8), the r-value reports effect sizes as small (0.1-<0.3), medium (0.3-<0.50) and large (>0.5). Small effect sizes are marked with italics, medium effect sizes are set using bold text and large effect sizes are marked with italics and bold text. HC sample and MS sample matched on age and sex based on available MS patients with 3T scan performed at time point 3.

**Case control: HC (n=235) vs MS**	**1.5 T**								
Brain age gaps residualized for age, age^2, sex and scanner	**Time point 1 (n=73)**								
Brain regions	*BAG*	*t*	*p*	*Cohen´s D (mean)*	*Cohen´s D (median)*	*Cohen´s D (rank)*	*MWU p-value*	*r*
Full brain	2.8	3.1	1.8×10^{-3}	*0.40*	*0.39*	*0.29*	0.03	*0.12*
Occipital	4.3	3.5	6.0×10^{-4}	*0.47*	*0.26*	*0.44*	1.2×10^{-3}	*0.19*
Temporal	-0.32	-0.02	0.99	0.04	0.14	0.10	0.45	0.04
Frontal	1.7	1.7	0.09	*0.21*	0.10	0.14	0.30	0.06
Parietal	0.4	0.8	0.45	0.06	0.16	0.04	0.75	0.02
Cingulate	4.5	3.4	8.2×10^{-4}	*0.43*	**0.50**	*0.43*	1.4×10^{-3}	*0.18*
Insula	4.0	3.1	2.2×10^{-3}	*0.42*	*0.26*	*0.41*	2.3×10^{-3}	*0.17*
Cerebellar / Subcortical	5.7	4.7	4.8×10^{-6}	**0.63**	***0.58***	**0.51**	2.0×10^{-4}	*0.21*
	**1.5 T**								
	**Time point 2 (n=58)**								
	BAG	*t*	*p*	*Cohen´s D (mean)*	*Cohen´s D (median)*	*Cohen´s D (rank)*	*MWU p-value*	*r*
Full brain	3.3	3.4	8.0×10^{-4}	*0.48*	*0.39*	*0.36*	0.01	*0.14*
Occipital	4.0	2.9	3.5×10^{-3}	*0.44*	*0.31*	*0.40*	6.6×10^{-3}	*0.16*
Temporal	0.02	0.13	0.90	0.00	0.01	0.02	0.91	0.01
Frontal	2.4	2.0	0.048	*0.28*	0.05	*0.20*	0.18	0.08
Parietal	0.9	1.1	0.28	0.13	0.04	0.08	0.57	0.04
Cingulate	4.0	2.8	5.9×10^{-4}	*0.38*	*0.48*	*0.36*	0.02	*0.14*
Insula	4.0	2.8	5.6×10^{-3}	*0.42*	*0.40*	*0.45*	2.7×10^{-3}	*0.18*
Cerebellar / Subcortical	6.5	4.9	1.9×10^{-6}	**0.71**	***0.80***	**0.55**	2.5×10^{-4}	*0.21*
	**1.5 T**								
	**Time point 3 (n=60)**								
	BAG	*t*	*p*	*Cohen´s D (mean)*	*Cohen´s D (median)*	*Cohen´s D (rank)*	*MWU p-value*	*r*
Full brain	4.6	4.5	1.1×10^{-5}	**0.64**	*0.48*	*0.46*	1.7×10^{-3}	*0.18*
Occipital	4.7	3.5	5.9×10^{-4}	**0.51**	*0.45*	*0.49*	8.8×10^{-4}	*0.19*
Temporal	0.63	0.5	0.62	0.07	0.03	0.03	0.85	0.01
Frontal	3.3	2.7	7.5×10^{-3}	*0.39*	*0.51*	*0.31*	0.03	*0.13*
Parietal	3.0	2.9	3.5×10^{-3}	*0.42*	*0.25*	*0.27*	0.06	*0.11*
Cingulate	5.5	3.6	3.9×10^{-4}	**0.52**	*0.63*	**0.52**	4.6×10^{-4}	*0.20*
Insula	4.8	3.5	6.1×10^{-4}	**0.51**	*0.44*	**0.54**	2.8×10^{-4}	*0.21*
Cerebellar / Subcortical	6.0	4.6	6.5×10^{-6}	**0.66**	***1.05***	**0.51**	4.9×10^{-4}	*0.20*
	**3 T**								
	**Time point 3 (n=58)**								
	BAG	*t*	*p*	*Cohen´s D (mean)*	*Cohen´s D (median)*	*Cohen´s D (rank)*	*MWU p-value*	*r*
Full brain	4.4	4.7	4.0×10^{-6}	**0.69**	***0.85***	**0.50**	2.2×10^{-5}	*0.25*
Occipital	4.3	3.2	1.4×10^{-3}	*0.48*	*0.35*	*0.37*	1.9×10^{-3}	*0.18*
Temporal	0.15	0.19	0.90	0.02	0.06	0.01	0.92	0.01
Frontal	2.9	2.4	0.019	*0.35*	*0.38*	*0.27*	0.02	*0.14*
Parietal	2.4	2.6	9.8×10^{-3}	*0.38*	*0.22*	*0.29*	0.02	*0.14*
Cingulate	5.1	3.2	1.3×10^{-3}	*0.48*	**0.63**	*0.39*	1.1×10^{-3}	*0.19*
Insula	4.3	3.0	2.6×10^{-3}	*0.45*	*0.48*	*0.37*	1.9×10^{-3}	*0.18*
Cerebellar / Subcortical	6.2	4.9	1.6×10^{-6}	**0.72**	**0.75**	**0.54**	5.3×10^{-5}	*0.27*

Supplementary table 5. Summary of predicted brain age data for global BAG in the longitudinal MS cohort. Brain age gaps were residualized for age, age^2, sex and scanner.

Longitudinal MS Cohort	
Time point 1	
Available MRI samples	*n=73*
Age, mean (SD)	35.3 (7.3)
Brain age gap, mean (SD)	5.5 (8.3)
Residualized brain age gap, mean (SD)	2.8 (9.0)
Time point 2	
Available MRI samples	*n=58*
Age, mean (SD)	36.2 (7.2)
Predicted brain age gap, mean (SD)	5.7 (9.2)
Residualized brain age gap, mean (SD)	3.3 (9.4)
Time point 3	
Available MRI samples	*n=60*
Age, mean (SD)	40.5 (7.3)
Predicted brain age gap, mean (SD)	5.4 (9.4)
Residualized brain age gap, mean (SD)	4.6 (9.8)
Rate of annual brain aging	
Available samples	*n=68*
Annual rate of brain aging, mean (SD)	0.02 (1.22)
Residualized annual rate of brain aging, mean (SD)	0.41 (1.23)

Supplementary table 6. Summary of the longitudinal brain aging in the longitudinal MS cohort. To calculate the One-sample t Test we used SPSS and used 0 as the test value compared to the annualized brain aging of the different brain regions

**Longitudinal MRI data (n=68)**		**One-sample t Test**	
Brain regions	*Rate of annual brain aging (years, (SD))*	*t*	*p*
Full brain	**0.41 (1.23)**	**2.76**	**0.008**
Occipital	-0.16 (1.38)	-0.95	0.35
Temporal	-0.09 (1.41)	-0.53	0.60
Frontal	0.36 (1.72)	1.73	0.09
Parietal	0.37 (1.59)	1.90	0.06
Cingulate	-0.07 (2.62)	-0.22	0.83
Insula	0.12 (2.02)	0.48	0.63
Cerebellar / Subcortical	0.35 (1.71)	1.71	0.09

Supplementary table 7. The global brain age gaps and correlation between the MRI data from the three processing steps at time point 1. We also listed the correlataions between chronologocal age and brain age gaps in the same three groups.

Effect of MRI postprocessing from time point 1	Raw images	Lesion filling	Longitudinal stream
Brain age gap (SD)	10.4 (8.7)	10.6 (8.9)	5.5 (8.3)
Residualized brain age gap, mean (SD)	2.5 (8.6)	2.7 (8.8)	-2.4 (8.1)
Correlation with chronological age, r	0.58	0.56	0.59
Correlation with raw images, r	-	0.98	0.96
Correlation with lesion filling, r	0.98	-	0.95
Correlation with FreeSurfer longitudinal stream, r	0.96	0.95	-

Supplementary table 8. Differences between the MS and HC samples across the three different processing steps at time point 1. Global brain age gaps (BAG) are residualized for age, age2, sex and scanner. Effect sizes were made using Cohen´s D estimates. HC sample and MS sample matched on age and sex based on available MS patients with 3T scan performed at time point 3.

**Case control: HC (n=235) vs MS (n=73)**	**1.5 T**											
Brain age gaps residualized for age, age^2, sex and scanner	***Time point 1 - Longitudinal Freesurfer***				***Time point 1 - Filled***				***Time point 1 - Raw***			
Brain regions	*BAG*	*t*	*p*	*Cohen´s D*	*BAG*	*t*	*p*	*Cohen´s D*	*BAG*	*t*	*p*	*Cohen´s D*
Full brain	2.8	3.1	1.8 x 10^-3	0.40	7.9	8.3	3.3 x 10^-15	1.11	7.7	8.1	1.4 x 10^-14	1.09
Occipital	4.3	3.5	6.0 x 10^-4	0.47	7.6	6.2	2.2 x 10^-9	0.82	8.2	6.4	4.4 x 10^-10	0.88
Temporal	-0.32	-0.02	0.99	-0.04	4.7	4.3	2.0 x 10^-5	0.62	5.1	4.7	2.2 x 10^-5	0.57
Frontal	1.7	1.7	0.09	0.21	6.8	6.0	4.8 x 10^-9	0.80	7.2	6.9	1.7 x 10^-9	0.84
Parietal	0.4	0.8	0.45	0.06	6.1	6.2	1.6 x 10^-9	0.82	6.6	6.2	2.4 x 10^-10	0.87
Cingulate	4.5	3.4	8.2 x 10^-4	0.43	12.0	8.7	2.8 x 10^-16	1.15	11.9	8.4	2.1 x 10^-15	1.13
Insula	4.0	3.1	2.2 x 10^-3	0.42	5.9	4.4	1.8 x 10^-5	0.61	5.5	5.6	6.4 x 10^-5	0.58
Cerebellar / Subcortical	5.7	4.7	4.8 x 10^-6	0.63	8.7	7.3	2.0 x 10^-12	1.01	8.5	8.3	2.7 x 10^-11	0.95

Supplementary table 9. An overview of the intraclass correlation coefficient (ICC), to show reliability between the brain age measures from the different time points, for all global and region wise brain age predictions before and after redisualization for age, age^2, sex and scanner.

**Predicted Age**	***Time point 1 vs. time point 2 (n=56 patients)***	***Time point 2 vs. time point 3 (n=49 patients)***	***Time point 1 vs. time point 3 (n=57 patients)***	***All time points(n=47 patients)***
Brain regions				
Full brain - ICC (95% CI)	0.96 (0.94-0.98)	0.87 (0.79-0.93)	0.83 (0.73-0.90)	0.89 (0.83-0.93)
Occipital - ICC (95% CI)	0.92 (0.86-0.95)	0.79 (0.66-0.88)	0.85 (0.76-0.91)	0.84 (0.75-0.90)
Temporal - ICC (95% CI)	0.93 (0.88-0.96)	0.86 (0.76-0.92)	0.87 (0.79-0.92)	0.88 (0.82-0.93)
Frontal - ICC (95% CI)	0.89 (0.81-0.93)	0.83 (0.71-0.90)	0.80 (0.68-0.88)	0.83 (0.74-0.89)
Parietal - ICC (95% CI)	0.91 (0.86-0.95)	0.76 (0.61-0.86)	0.76 (0.63-0.85)	0.79 (0.69-0.87)
Cingulate - ICC (95% CI)	0.89 (0.82-0.94)	0.78 (0.65-0.87)	0.77 (0.64-0.86)	0.83 (0.74-0.90)
Insula - ICC (95% CI)	0.89 (0.82-0.94)	0.76 (0.61-0.86)	0.80 (0.68-0.88)	0.82 (0.72-0.89)
Cerebellar / Subcortical - ICC (95% CI)	0.97 (0.96-0.99)	0.93 (0.88-0.96)	0.91 (0.85-0.94)	0.94 (0.91-0.97)
**Residualized brain age gap**				
Full brain - ICC (95% CI)	0.94 (0.90-0.96)	0.83 (0.72-0.90)	0.88 (0.8-0.93)	0.87 (0.81-0.92)
Occipital - ICC (95% CI)	0.92 (0.86-0.95	0.79 (0.66-0.88)	0.87 (0.79-0.92)	0.84 (0.76-0.90
Temporal - ICC (95% CI)	0.92 (0.86-0.95)	0.85 (0.75-0.91)	0.90 (0.83-0.94)	0.89 (0.82-0.93)
Frontal - ICC (95% CI)	0.87 (0.79-0.92)	0.82 (0.70-0.89)	0.84 (0.74-0.90)	0.84 (0.76-0.90)
Parietal - ICC (95% CI)	0.89 (0.83-0.94)	0.73 (0.57-0.84)	0.78 (0.65-0.86)	0.78 (0.67-0.86)
Cingulate - ICC (95% CI)	0.87 (0.79-0.92)	0.79 (0.66-0.88)	0.79 (0.66-0.87)	0.83 (0.75-0.90)
Insula - ICC (95% CI)	0.88 (0.81-0.93)	0.75 (0.60-0.85)	0.82 (0.72-0.89)	0.83 (0.75-0.90)
Cerebellar / Subcortical - ICC (95% CI)	0.98 (0.96-0.99)	0.93 (0.88-0.96)	0.92 (0.87-0.95)	0.95 (0.92-0.97)

Supplementary table 10. Pearson´s correlations between annualized brain aging and relevant clinical and MRI variables. Significant associations are highlighted with bold ($p<0.05$). Associations which were still significant after adjusting for false discovery rate are highlighted in red. Abbreviations: Expanded Disability Status Scale (EDSS), Multiple Sclerosis Severity Score (MSSS), Timed 25 Feet Walk Test (T25FWT), Nine hole peg test (9HPT), Disease-modifying therapies (DMT), Fatigue Severity Score (FSS), Beck Depression Index (BDI), Oligoclonal Bands (OBC), No Evidence of Disease Activity (NEDA), White Matter Lesion Load (WMLL) and Intracranial Volumne (ICV).

Pearson´s correlations with annual rate of brain aging on time point 3

	Full brain		*Occipital*		*Temporal*		*Frontal*		*Parietal*		*Cingulate*		*Insula*		*Cereb. / Subcort.*	
Clinical variables	*cor*	*p*	*cor*	*p*	*cor*	*p*	*cor*	*p*	*cor*	*p*	*cor*	*p*	*cor*	*p*	*cor*	*p*
EDSS	0.09	0.49	**-0.27**	**0.034**	-0.02	0.88	-0.01	0.95	-0.15	0.25	**-0.28**	**0.028**	-0.17	0.18	0.22	0.08
Change in EDSS	0.16	0.23	-0.15	0.26	-0.01	0.94	0.09	0.50	-0.03	0.83	-0.19	0.14	-0.13	0.34	**0.29**	**0.026**
MSSS	-0.03	0.84	-0.24	0.06	-0.18	0.17	-0.09	0.47	-0.21	0.11	**-0.32**	**0.011**	-0.19	0.14	0.17	0.20
Change in MSSS	0.17	0.21	-0.04	0.75	-0.01	0.94	0.10	0.46	0.05	0.68	-0.11	0.39	-0.05	0.70	**0.36**	**5.1 x 10^-3**
T25FWT	0.06	0.63	-0.13	0.33	0.08	0.56	0.06	0.66	-0.01	0.95	-0.12	0.36	-0.01	0.92	0.01	0.96
Change in T25FWT	-0.03	0.85	-0.09	0.50	0.01	0.96	-0.01	0.97	0.02	0.86	-0.21	0.11	0.02	0.86	0.08	0.56
9HPT Dominant	0.11	0.40	-0.09	0.47	0.01	0.92	0.02	0.91	-0.15	0.25	-0.23	0.07	-0.08	0.56	0.06	0.64
Change in 9HPT Dominant	0.11	0.38	-0.07	0.62	0.05	0.70	0.07	0.60	-0.10	0.45	-0.13	0.31	-0.07	0.62	0.00	0.99
9HPT Non-dominant	**0.29**	**0.028**	-0.06	0.66	0.18	0.18	0.15	0.27	0.01	0.92	-0.10	0.45	0.04	0.76	**0.30**	**0.021**
Change in 9HPT Non-dominant	**0.31**	**0.017**	0.00	0.98	0.13	0.33	0.20	0.14	0.08	0.53	-0.06	0.63	0.00	0.97	**0.32**	**0.014**
DMT Level	**-0.28**	**0.031**	-0.15	0.27	-0.24	0.06	-0.22	0.09	-0.17	0.21	-0.11	0.39	-0.13	0.31	-0.08	0.54
Total attacks	-0.22	0.10	**-0.26**	**0.043**	-0.23	0.08	-0.12	0.35	-0.24	0.07	-0.22	0.10	**-0.27**	**0.033**	0.16	0.21
New attacks	-0.09	0.50	-0.14	0.30	-0.10	0.47	-0.13	0.32	-0.11	0.41	-0.08	0.52	-0.16	0.23	0.03	0.83
OCB status	-0.16	0.20	-0.01	0.93	-0.23	0.06	-0.02	0.87	-0.02	0.89	0.05	0.66	-0.06	0.62	-0.17	0.17
Gender	-0.05	0.66	0.04	0.74	-0.08	0.54	0.06	0.60	0.04	0.74	0.02	0.90	0.17	0.17	-0.11	0.37
NEDA-3	-0.02	0.90	-0.08	0.56	-0.08	0.54	0.05	0.70	-0.06	0.66	-0.04	0.78	-0.12	0.38	0.11	0.40
NEDA-4	0.06	0.65	0.02	0.86	-0.02	0.86	0.12	0.37	0.01	0.97	-0.06	0.66	0.00	1.00	0.15	0..28
Distance from diagnosis	-0.04	0.74	-0.15	0.25	0.07	0.60	-0.15	0.26	-0.05	0.68	0.01	0.92	-0.09	0.51	0.00	0.98
Distance from first symptom	-0.06	0.66	-0.10	0.43	0.09	0.49	-0.09	0.50	-0.01	0.93	-0.07	0.58	-0.11	0.39	-0.15	0.26
MRI variables	*cor*	*p*	*cor*	*p*	*cor*	*p*	*cor*	*p*	*cor*	*p*	*cor*	*p*	*cor*	*p*	*cor*	*p*
WMLL	**0.29**	**0.026**	0.13	0.33	**0.29**	**0.027**	0.21	0.11	0.19	0.16	-0.02	0.90	-0.01	0.94	0.01	0.96
Change in WMLL	**0.30**	**0.015**	**0.34**	**4.9 x 10^-3**	**0.34**	**5.7 x 10^-3**	0.19	0.12	**0.35**	**4.3 x 10^-3**	0.10	0.43	0.14	0.27	0.00	0.98
Brain volume	-0.01	0.93	-0.02	0.89	-0.11	0.41	-0.08	0.54	-0.03	0.83	-0.04	0.75	0.01	0.95	0.10	0.44
Brain atrophy	**-0.79**	**4.3 x 10^-15**	**-0.39**	**1.2 x 10^-3**	**-0.74**	**1.6 x 10^-12**	**-0.79**	**1.6 x 10^-15**	**-0.72**	**1.1 x 10^-11**	**-0.35**	**3.9 x 10^-3**	**-0.65**	**2.1 x 10^-8**	-0.07	0.57
ICV	0.01	0.95	-0.04	0.75	-0.05	0.71	-0.07	0.58	-0.05	0.67	0.04	0.73	-0.12	0.34	-0.01	0.94

Supplementary table 11. Pearson´s correlations between brain age gap and relevant clinical and MRI variables. Significant associations are highlighted with bold ($p<0.05$). Associations which were still significant after adjusting for false discovery rate are highlighted in red. Abbreviations: Expanded Disability Status Scale (EDSS), Multiple Sclerosis Severity Score (MSSS), Timed 25 Feet Walk Test (T25FWT), Nine hole peg test (9HPT), Disease-modifying therapies (DMT), Fatigue Severity Score (FSS), Beck Depression Index (BDI), Oligoclonal Bands (OBC), No Evidence of Disease Activity (NEDA), White Matter Lesion Load (WMLL) and Intracranial Volumne (ICV).

Pearson´s correlations with brain age gap on time point 3

	Full brain		*Occipital*		*Temporal*		*Frontal*	
Clinical variables	*cor*	*p*	*cor*	*p*	*cor*	*p*	*cor*	*p*
EDSS	0.11	0.39	-0.10	0.44	0.11	0.40	-0.05	0.73
Change in EDSS	0.05	0.69	-0.11	0.39	0.07	0.61	-0.15	0.27
MSSS	-0.04	0.73	-0.14	0.28	-0.04	0.79	-0.15	0.26
Change in MSSS	-0.04	0.78	0.02	0.86	0.08	0.56	-0.20	0.13
T25FWT	0.12	0.35	-0.10	0.46	0.14	0.28	-0.03	0.82
Change in T25FWT	0.04	0.74	-0.03	0.84	0.03	0.82	-0.19	0.15
9HPT Dominant	0.16	0.23	-0.17	0.19	0.01	0.93	-0.10	0.46
Change in 9HPT Dominant	0.05	0.70	-0.15	0.27	-0.01	0.93	-0.12	0.36
9HPT Non-dominant	**0.36**	**5.8 x 10^-3**	-0.11	0.42	0.16	0.22	0.03	0.80
Change in 9HPT Non-dominant	**0.28**	**0.035**	-0.19	0.14	0.15	0.28	0.05	0.68
DMT Level	0.01	0.93	-0.11	0.40	0.01	0.93	0.03	0.80
Total attacks	-0.20	0.13	-0.16	0.23	-0.05	0.70	-0.14	0.30
New attacks	-0.17	0.20	-0.23	0.08	-0.12	0.37	-0.20	0.13
OCB status	-0.04	0.75	-0.03	0.83	-0.16	0.22	0.00	0.98
Gender	**-0.28**	**0.031**	-0.06	0.65	-0.21	0.11	0.05	0.68
NEDA-3	-0.16	0.22	-0.01	0.94	-0.05	0.72	-0.13	0.31
NEDA-4	-0.08	0.55	0.00	0.99	0.08	0.55	-0.05	0.72
Distance from diagnosis	-0.21	0.10	0.04	0.78	0.00	0.98	-0.15	0.25
Distance from first symptom	0.18	0.17	0.01	0.92	0.00	1.00	-0.05	0.69
MRI variables	*cor*	*p*	*cor*	*p*	*cor*	*p*	*cor*	*p*
WMLL	**0.46**	**3.0 x 10^-4**	0.01	0.95	**0.30**	**0.025**	0.19	0.16
Change in WMLL	**0.30**	**0.022**	0.02	0.89	**0.30**	**0.025**	0.12	0.34
Brain volume	-0.25	0.06	**-0.38**	**3.2 x 10^-3**	**-0.32**	**0.016**	**-0.43**	**8.8 x 10^-4**
Brain atrophy	**-0.33**	**0.011**	-0.20	0.14	**-0.41**	**1.6 x 10^-3**	**-0.31**	**0.017**
ICV	-0.01	0.94	**-0.31**	**0.019**	-0.22	0.09	**-0.29**	**0.027**

	Parietal		*Cingulate*		*Insula*		*Cereb. / Subcort.*	
Clinical variables	*cor*	*p*	*cor*	*p*	*cor*	*p*	*cor*	*p*
EDSS	0.05	0.68	-0.22	0.09	-0.11	0.42	0.09	0.51
Change in EDSS	0.03	0.83	-0.16	0.22	0.01	0.91	-0.01	0.96
MSSS	-0.09	0.48	**-0.27**	**0.039**	**-0.29**	**0.025**	0.00	0.98
Change in MSSS	-0.02	0.89	-0.04	0.78	0.06	0.62	-0.11	0.42
T25FWT	0.01	0.95	-0.13	0.33	0.03	0.84	0.18	0.17
Change in T25FWT	0.02	0.89	-0.02	0.85	0.12	0.35	0.10	0.45
9HPT Dominant	-0.03	0.83	-0.25	0.055	-0.04	0.75	0.04	0.78
Change in 9HPT Dominant	-0.07	0.58	-0.14	0.27	-0.10	0.43	-0.01	0.95
9HPT Non-dominant	0.16	0.22	-0.22	0.10	0.12	0.37	**0.28**	**0.030**
Change in 9HPT Non-dominant	0.14	0.31	-0.09	0.50	0.14	0.28	0.21	0.12
DMT Level	-0.05	0.70	0.04	0.79	-0.01	0.93	**0.26**	**0.046**
Total attacks	-0.16	0.21	0.00	0.99	**-0.26**	**0.047**	0.03	0.84
New attacks	-0.22	0.08	-0.03	0.81	-0.13	0.31	0.09	0.49
OCB status	-0.11	0.42	0.03	0.82	-0.01	0.95	-0.03	0.82
Gender	-0.18	0.17	**0.26**	**0.046**	**-0.30**	**0.021**	-0.04	0.78
NEDA-3	-0.13	0.31	-0.11	0.39	-0.22	0.08	-0.04	0.78
NEDA-4	-0.10	0.49	-0.11	0.41	-0.18	0.19	0.00	0.99
Distance from diagnosis	-0.10	0.47	0.01	0.95	-0.21	0.12	-0.18	0.16
Distance from first symptom	0.14	0.27	0.01	0.92	0.13	0.31	0.18	0.16
MRI variables	*cor*	*p*	*cor*	*p*	*cor*	*p*	*cor*	*p*
WMLL	0.24	0.07	0.04	0.77	0.23	0.08	**0.38**	**3.2 x 10^-3**
Change in WMLL	0.20	0.13	0.06	0.64	0.14	0.29	**0.34**	**9.6 x 10^-3**
Brain volume	**-0.35**	**7.3 x 10^-3**	**-0.40**	**2.2 x 10^-3**	-0.05	0.70	-0.24	0.07
Brain atrophy	**-0.37**	**4.7 x 10^-3**	**-0.28**	**0.032**	**-0.33**	**0.013**	-0.13	0.32
ICV	-0.20	0.13	**-0.29**	**0.027**	0.02	0.86	-0.02	0.87

Supplementary table 12. Pearson´s correlations between differenct MRI variables and EDSS and MSSS. Significant associations are highlighted with bold (p<0.05), no assiciations were still significant after adjusting for false discovery rate. Abbreviations: Expanded Disability Status Scale (EDSS), Multiple Sclerosis Severity Score (MSSS), White Matter (WM), Grey Matter (GM).

	**Pearson´s correlations with MRI variables**							
	EDSS		*Change in EDSS*		*MSSS*		*Change in MSSS*	
MRI variables	*cor*	*p*	*cor*	*p*	*cor*	*p*	*cor*	*p*
Full brain brain age gap	0.11	0.39	0.05	0.69	-0.04	0.74	-0.04	0.78
Cereb. / Subcort. brain age gap	*0.09*	0.51	-0.01	0.96	0.00	0.98	-0.11	0.42
Brain aging full brain	*0.09*	0.49	0.16	0.23	-0.03	0.84	0.17	0.21
Brain aging cereb. / Subcort.	*0.22*	0.08	**0.29**	**0.03**	0.17	0.20	**0.36**	**5.1×10^{3}**
Brain volume 1.5T	*-0.05*	0.71	0.20	0.13	0.03	0.82	0.14	0.28
Brain volume 3T	*-0.07*	0.61	0.18	0.17	-0.05	0.70	0.13	0.38
Brain atrophy 1.5T	*-0.05*	0.68	0.02	0.89	0.11	0.39	0.03	0.80
WM volume 1.5T	*0.02*	0.86	**0.25**	**0.05**	-0.01	0.92	0.13	0.35
WM volume 3T	0.01	0.93	0.25	0.06	-0.03	0.81	0.13	0.35
GM volume 1.5T	*-0.13*	0.34	0.12	0.37	-0.05	0.70	0.15	0.28
GM volume 3T	*-0.15*	0.27	0.09	0.50	-0.07	0.59	0.12	0.39
Normalised brain volume 1.5T	*0.01*	0.97	0.02	0.89	0.07	0.58	0.12	0.36
Normalised brain volume 3T	*-0.16*	0.22	-0.17	0.21	-0.11	0.39	0.02	0.88
Normalised WM volume 1.5T	*0.13*	0.33	0.20	0.13	0.01	0.92	0.11	0.42
Normalised WM volume 3T	0.02	0.89	0.11	0.42	-0.03	0.81	0.07	0.60
Normalised GM volume 1.5T	*-0.11*	0.42	-0.14	0.28	0.03	0.83	0.07	0.58
Normalised GM volume 3T	*-0.22*	0.09	0.29	0.03	-0.10	0.44	0.07	0.59

Supplementary table 13. Group characteristics based on the DMT level at time point 3, also taking into account retrospective DMT switches. DMT level is explained in the methods section. Three MS patients didn´t fit these groups and were excluded in this table. Abbreviations: Expanded Disability Status Scale (EDSS), Timed 25 Feet Walk Test (T25FWT), Multiple Sclerosis Severity Score (MSSS), Fatigue Severity Score (FSS) and Beck Depression Index (BDI).

	**Disease-modifying therapies**		
**Variables**	**No treatment, n=21**	**First line, n=30**	**Second line, n=22**
Age MS Onset (SD)	31.4 (6.6)	29.0 (6.2)	27.6 (7.1)
Female, n (%)	15 (71)	19 (63)	17 (77)
EDSS time point 1, mean (SD)	2.3 (1.2)	1.8 (0.8)	2.0 (0.7)
EDSS time point 2, mean (SD)	2.4 (1.0)	1.7 (0.9)	1.8 (0.7)
EDSS time point 3, mean (SD)	2.2 (1.6)	2.1 (1.1)	1.7 (1.0)
EDSS change, mean (SD)	0.03 (1.1)	0.2 (0.8)	-0.4 (0.9)
Brain age gap time point 1, mean (SD)	-2.8 (9.3)	-1.7 (8.3)	-1.7 (9.3)
Brain age gap time point 2, mean (SD)	-1.4 (10.1)	-2.6 (7.5)	-0.7 (10.1)
Brain age gap time point 3, mean (SD)	0.2 (9.1)	-0.7 (9.6)	0.2 (10.3)
Annual rate of brain aging, mean (SD)	0.92 (0.82)	0.13 (1.3)	0.35 (1.3)
T25FWT time point 3, mean seconds (SD)	4.2 (1.9)	3.9 (0.6)	4.0 (0.6)
T25FWT change, mean seconds (SD)	0.2 (1.4)	0.0 (1.1)	0.1 (0.5)
MSSS Change, mean (SD)	-1.8 (1.3)	-2.0 (1.7)	-3.1 (2.1)
Disease duration time point 3, mean years (SD)	11.6 (5.7)	11.2 (5.9)	8.7 (4.2)
